# Digital PCR outperforms quantitative real-time PCR for the detection and quantification of major periodontal pathobionts

**DOI:** 10.1080/20002297.2025.2537439

**Published:** 2025-07-23

**Authors:** Haris Munjaković, Katja Povšič, Mario Poljak, Katja Seme, Rok Gašperšič, Lucijan Skubic

**Affiliations:** a Department of Oral Medicine and Periodontology, University Medical Centre Ljubljana, Ljubljana, Slovenia; b Department of Oral Medicine and Periodontology, Faculty of Medicine, University of Ljubljana, Ljubljana, Slovenia; c Institute of Microbiology and Immunology, Faculty of Medicine, University of Ljubljana, Ljubljana, Slovenia; d Clinical Institute for Special Laboratory Diagnostics, University Medical Centre Ljubljana, Ljubljana, Slovenia

**Keywords:** Digital PCR, oral microbiology, periodontal disease, quantitative real-time PCR, subgingival plaque

## Abstract

**Background:**

This study comparatively evaluated the analytical and diagnostic performance of a multiplex digital polymerase-chain reaction (dPCR) assay and a quantitative real-time PCR (qPCR) for the simultaneous detection and quantification of periodontal pathobionts: *Porphyromonas gingivalis*, *Aggregatibacter actinomycetemcomitans*, and *Fusobacterium nucleatum*.

**Materials and Methods:**

Subgingival plaque samples from 20 periodontitis patients and 20 periodontally healthy controls were analyzed. Several analytical parameters of the dPCR assay, optimized using DNA standards, were assessed versus qPCR: dynamic range linearity, precision, accuracy, prevalence, sensitivity, specificity, and concordance. The statistical analyses included Mann-Whitney U test, Wilcoxon test, McNemar’s test, and Bland-Altman plots.

**Results:**

dPCR showed high linearity (R^2^ > 0.99) and lower intra-assay variability (median CV%: 4.5%) than qPCR (*p* = 0.020), with comparable accuracy and agreement. dPCR demonstrated superior sensitivity, detecting lower bacterial loads, particularly for *P.* *gingivalis* and *A.* *actinomycetemcomitans*. The Bland-Altman plots highlighted good agreement at medium/high loads but discrepancies at low concentrations (< 3 log_10_Geq/mL), resulting in qPCR false negatives and a 5-fold underestimation of the prevalence of *A.* *actinomycetemcomitans* in periodontitis patients. High concordance between the assays was observed for *F.* *nucleatum* across both study groups.

**Conclusions:**

dPCR outperformed qPCR for quantifying periodontal pathobionts and had superior sensitivity and precision, making it particularly effective in detecting low-level bacterial loads.

## Introduction

Periodontal disease is a highly individualized condition induced by distinct host–microbiota interactions [1]. In healthy periodontal tissues, a resilient microbiota within the subgingival biofilm sustains a symbiotic relationship with a host [2]. Various factors can disrupt this balance, resulting in significant phenotypic biofilm alterations [3]. These dysbiotic changes not only undermine the microbiota’s resilience [4,5] but also trigger host immune-inflammatory responses and periodontal breakdown [6].

**Figure 1.**
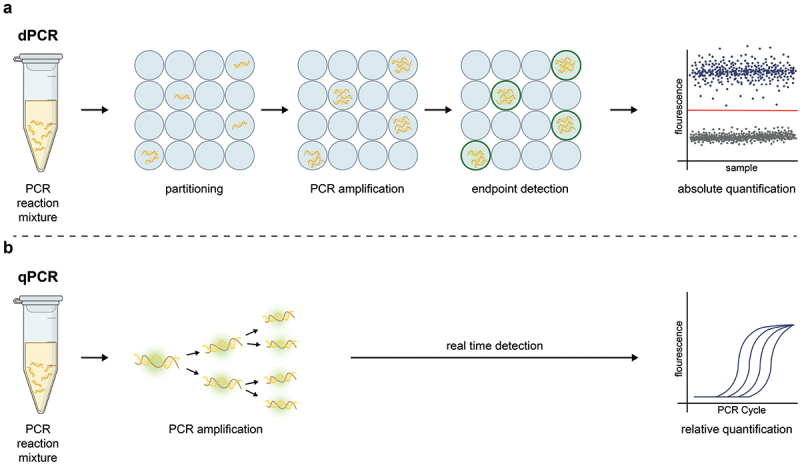
Digital PCR and quantitative real-time PCR workflow diagram.

Molecular diagnostics have a pivotal role in both basic science and clinical periodontal research [7]. Digital polymerase-chain reaction (dPCR) is becoming increasingly recognized among molecular methods in the field of microbiology since it enables the detection and absolute quantification of target nucleic acids (DNA or RNA) with a high degree of sensitivity and precision [8,9]. Compared to quantitative real-time PCR (qPCR), the entire volume of the dPCR reaction mixture containing template nucleic acids is randomly and independently divided into several thousands of smaller partitions, in which the amplification of target nucleic acids occurs separately, thereby minimising competition between the targets (Figure 1). A fluorescence signal is subsequently measured after the reaction is completed (i.e. endpoint detection). dPCR does not require the generation of a calibration curve for quantification, allowing for higher tolerances to inhibitors and greater robustness to changes in amplification [9,10].

In digital PCR (dPCR) (a), the reaction mixture is divided into thousands of partitions, each of which undergoes separate PCR amplification of the target nucleic acids. The fluorescence signal of each partition is measured at the end of PCR amplification, and the partitions are classified as positive (blue) or negative (grey) based on their fluorescence signals. In contrast, in quantitative real-time PCR (qPCR) (b), the reaction mixture is amplified as a whole, and the fluorescence signal is measured in real time, at the end of each PCR amplification cycle.

Previous studies have used dPCR to detect *Fusobacterium nucleatum* in oral squamous cell carcinoma [11], colorectal cancer research [12–14], and forensic microbiology [15], as well as to investigate the influence of *Porphyromonas gingivalis* on preterm delivery [16]. Despite an extensive review of the published literature, no studies to date have reported the use of dPCR for the simultaneous detection and quantification of multiple oral bacteria for epidemiological characterization in clinical periodontal samples.

The aim of this study was to detect and quantify the three most studied putative periodontal pathobionts [17–19]: *P. gingivalis*, *Aggregatibacter actinomycetemcomitans*, and *F. nucleatum* in subgingival plaque samples of periodontitis patients and healthy controls, using a newly developed multiplex dPCR assay in comparison to the canonical qPCR method. Compared to qPCR, the partitioning-based principle of dPCR improves precision, suitability for multiplex analyses, and the detection of low abundant targets within a high background of other target sequences in complex clinical samples [8,9]. The dPCR approach may thus be particularly effective for the detection of extremely low bacterial concentrations, providing insights into the potential early dynamics of subgingival plaque colonization in periodontitis and, even more so at periodontally healthy sites.

## Materials and methods

The samples used in the study were collected from November−December 2024 at the Department of Oral Medicine and Periodontology, University Dental Clinic of Ljubljana, Slovenia. The study was approved by the National Medical Ethic on 28 August 2024 (0120–444/2022/3) and registered at Clintrials.gov (NCT06692595). All participants signed an informed consent form. The principles of the Declaration of Helsinki were followed.

### Study population

The study comprised of two subject groups. The first group consisted of 20 out of 78 consecutively screened subjects with periodontitis (convenience sample), who had at least one probing site in each jaw quadrant with pocket depth (PD) measuring > 4 mm and positive bleeding on probing (BOP). Periodontitis stage and grade were determined following the 2018 classification system [20,21]. The second group consisted of 20 out of 38 consecutively screened volunteers without periodontal pathologies (healthy group), i.e. plaque index < 10% at baseline visit, absence of periodontal pockets measuring ≥4 mm and no prior periodontal treatment. Inclusion and exclusion criteria for the study population are provided in Supplementary Table S1.

### Clinical examination and microbiological sampling

A baseline full periodontal examination was performed prior to subject inclusion by an experienced calibrated examiner (R. G.) [21]. The presence of plaque (PlI) using O’Leary’s dichotomous Plaque Control Record [22], PD, gingival recession (REC) and BOP were assessed at 6 sites per tooth using a manual Williams probe (POW6, Hu-Friedy, Chicago, Illinois, USA). Clinical attachment loss (CAL) was calculated as a sum of REC and PD. Furcation involvement and tooth mobility were assessed at each tooth.

Samples of subgingival plaque were collected at four sites per subject to obtain a pooled sample. In the periodontitis group, one site with the deepest PD and positive BOP was selected in each jaw quadrant, while four Ramfjord teeth (16, 21, 36, 41) were selected for sampling in the healthy group. Sampling sites were cleansed of supragingival plaque using cotton pellets and then dried using cotton rolls and gentle air drying. Two absorbent paper points (0.30 mm in diameter, Maillefer, Ballaigues, Switzerland) were inserted into the selected periodontal pockets for 10 s and pooled into sample tubes containing 1 mL of reduced transport fluid (RTF) with 10% glycerol. The samples were immediately stored at −20°C.

Subjects with periodontitis were then treated in accordance with the 2020 treatment guidelines [23].

### Bacterial reference strains

The reference strains *P. gingivalis* ATCC 33277, *F. nucleatum* subsp. *polymorphum* DSM 20482, and *A. actinomycetemcomitans* ATCC 43718 were included in this study. All strains were maintained as described in the Supplementary Appendix.

### DNA extraction

DNA was extracted using the QIAamp DNA Mini kit (Qiagen, Hilden, Germany) following the manufacturer’s instructions [24]. A detailed description of DNA extraction is provided in the Supplementary Appendix.

### Primers and probes

Oligonucleotide primers and double-quenched hydrolysis probes based on 16S rRNA genes targeting *P. gingivalis*, *A. actinomycetemcomitans*, and *F. nucleatum* sequences, along with details, are provided in the Supplementary Appendix and Supplementary Table S2.

### Digital PCR (dPCR) assay

To determine the presence of *P. gingivalis*, *A. actinomycetemcomitans*, and *F. nucleatum* in samples, nanoplate-based microfluidic multiplex dPCR assays were performed using the QIAcuity Probe PCR Kit (Qiagen) in 40 μL reaction mixtures, each containing 10 µL of sample DNA, 10 µL of 4× Probe PCR Master Mix, 0.4 µM of each of the specific primers, 0.2 µM of each of the specific probes (Supplementary Table S2), 0.025 U/µL of the restriction enzyme Anza 52 *Pvu*II (Thermo Scientific, Waltham, MA, USA), and nuclease-free water. The reaction mixtures were prepared in pre-plates, transferred to the QIAcuity Nanoplate 26k 24-well plate (Qiagen), sealed with the QIAcuity Nanoplate Seal (Qiagen), and then loaded into the automated dPCR instrument QIAcuity Four (Qiagen), as instructed by the manufacturer. The workflow included: (i) a priming and rolling step with partitioning of the reaction mixture in each well into approximately 26.000 partitions, (ii) a thermocycling step under the following conditions: initial DNA denaturation and enzyme activation for 2 min at 95°C, followed by 45 amplification cycles of 15 s at 95°C and 1 min at 58°C, and (iii) an imaging step with image acquisition (i.e. determination of positive and negative partitions) on the green channel (target: *A. actinomycetemcomitans*, threshold: 30 RFU, exposure time: 500 ms, gain: 6 dB), yellow channel (target: *P. gingivalis*, threshold: 40 RFU, exposure time: 500 ms, gain: 6 dB), and crimson channel (target: *F. nucleatum*, threshold: 40 RFU, exposure time: 400 ms, gain: 8 dB), respectively. Data were analyzed using the QIAcuity Software Suite v2.5.0.1 (Qiagen) and DNA concentrations were automatically calculated according to the Poisson distribution principle. To improve the accuracy of the concentration calculation in each well, the Volume Precision Factor v9.0 (Qiagen) was applied according to the manufacturer’s instructions. A dPCR reaction was considered positive if at least three partitions were positive. The optimization data for the multiplex dPCR assay, including details of the tested template DNA dilutions, annealing temperatures, and primer-probe concentrations, can be found in Supplementary Figures S1 − 4.

For samples where the qPCR results indicated an excessive concentration of bacterial target DNA (i.e. > 10^5^ copies of target DNA per reaction), two consecutive 10-fold dilutions of the original DNA isolates were tested to avoid positive fluorescence signal saturation, which would otherwise lead to an underestimation of the sample template DNA concentration in dPCR assays [25]. The average values of the measurements in the tested dilutions were subsequently used to determine the DNA concentration in the original samples. All 10-fold serial dilutions were prepared manually in 1.5 mL DNA LoBind tubes (Eppendorf, Hamburg, Germany) using a water solution with carrier RNA (1 μg/mL) (Qiagen). Exemplary one-dimensional scatter plots showing the results of total DNA isolates and dilutions of the clinical samples with the multiplex dPCR assay are shown in the Supplementary Figure S5.

Human genomic DNA (Roche Diagnostics, Mannheim, Germany) was used as a control to demonstrate the absence of cross-reactivity of the primer-probe sets with human DNA. Human genomic DNA was tested with multiplex dPCR assays, as described above, and singleplex dPCR assays containing only primer-probe sets for particular bacterial species. A reaction mixture with nuclease-free water instead of the DNA template (no-template control) was used in all dPCR runs as a reference control for potential amplicon carryover contamination.

### Quantitative real-time PCR (qPCR) assay

A multiplex qPCR assay targeting *P. gingivalis*, *A. actinomycetemcomitans*, and *F. nucleatum* was conducted on a QuantStudio 7 Pro instrument (Applied Biosystems, Life Technologies, Carlsbad, CA, USA). Fluorescence acquisition occurred at the elongation step of each cycle. Bacterial DNA concentrations were determined via a standard curve method. A detailed description of the multiplex qPCR assay is provided in the Supplementary Appendix.

### Analytical validation

The analytical properties of the dPCR and qPCR methods were verified by testing triplicates of 10-fold serially diluted standards of bacterial target DNA, corresponding to the estimated input concentration range of 1 × 10^6^ to 1 × 10^−1^ copies of target DNA per reaction. Total DNA isolates of the bacterial reference strains *P. gingivalis*, *A. actinomycetemcomitans*, and *F. nucleatum* were used as standards. The dilution series were tested in multiplex dPCR and qPCR assays, as described above, as well as in singleplex dPCR and qPCR assays, containing only primer-probe sets for particular bacterial species.

### Statistics

The primary quantitative outcome variable for both methods was the concentration of targeted bacterial species, expressed as genome equivalents per mL (Geq/mL), normalized for ribosomal gene copy number per bacteria. The coefficients of determination (R^2^) of the linear regression lines between the logarithm of the measured concentrations of bacterial target DNA in serially diluted standards by the multiplex qPCR and dPCR assays and the logarithm of the estimated concentrations of bacterial DNA were used to determine the linearity over the dynamic range. In addition, the precision (intra-assay variability) of the quantification of bacterial DNA in serially diluted standards was determined for both assays by evaluating the coefficient of variation percentage (CV%) [26]. The accuracy (trueness) of both assays was calculated as the closeness of agreement between the mean values of the concentration measurements in serially diluted standards and the estimated values [27]. The Wilcoxon matched-pairs signed-rank test was used to compare paired bacterial DNA concentration measurements in clinical samples. The McNemar's test was used to compare frequency distributions between qPCR and dPCR outcomes in paired clinical samples; a *post-hoc* power analysis was performed thereafter.

Descriptive statistics included the mean values with standard deviations (SD) and median values with interquartile ranges (IQR), while patient-related secondary outcomes were the prevalence of targeted pathobionts and clinical parameters (PD, REC, BOP, CAL, PII) expressed as means and SD. After evaluating the normality of the distribution using the Shapiro–Wilk test, a Mann-Whitney *U* statistical test was performed. Bland-Altman tests were used to assess the agreement between qPCR and dPCR measurements in clinical samples, expressed as quantification bias with SD and 95% limits of agreement. Quantitative diagnostic analytic parameters (sensitivity, specificity, positive predictive value, and negative predictive value) were calculated using both multiplex qPCR and dPCR assays as reference methods. Additionally, Lin’s concordance correlation coefficient was assessed. Statistical analyses were performed with GraphPad Prism 10.1.2 Software (GraphPad Software, San Diego, California, USA), and MedCalc Software (Ostend, Belgium), with a confidence level of 95%.

## Results

### Study population

Demographic and clinical data are shown in Supplementary Table S4 and S5.

### Analytical performance

Testing triplicates of 10-fold serially diluted standards of *P. gingivalis*, *A. actinomycetemcomitans*, and *F. nucleatum*, corresponding to the estimated input concentration range of 1 × 10^6^ to 1 × 10^−1^ copies of target DNA per reaction, demonstrated that multiplex dPCR and qPCR assays had a sensitivity of at least 10 copies of target DNA per reaction (Supplementary Figure S6, Supplementary Table S6). Both multiplex dPCR and qPCR assays confirmed a high degree of linearity over the dynamic range from 1 × 10^5^ to 1 × 10^1^ copies of target DNA per reaction. At a target concentration of 1 × 10^6^ copies per reaction, positive saturation of the fluorescence signal was observed in the partitions of dPCR (Supplementary Figure S7), while qPCR assays maintained linearity. When targeting all the tested bacteria, the R^2^ values of both assays were > 0.99 (Supplementary Figure S6). Pearson’s correlation coefficient confirmed that both multiplex dPCR and qPCR assays showed a very high positive correlation for the quantification of bacterial target DNA in standard dilutions (*r* = 0.995; *p* < 0.0001).

**Figure 2.**
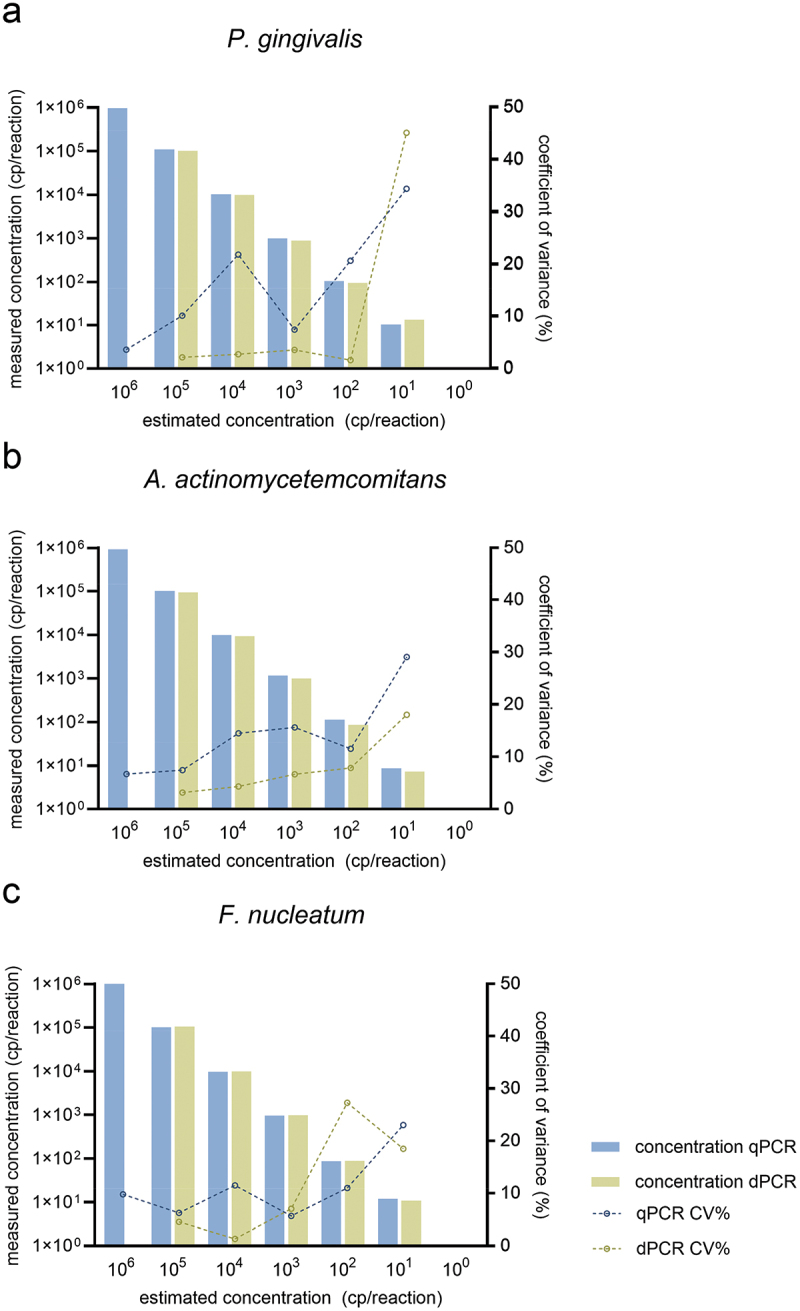
Precision of quantification of bacterial target DNA in the serially diluted standards of *Porphyromonas gingivalis*, *Aggregatibacter actinomycetemcomitans*, and *Fusobacterium nucleatum* by the multiplex quantitative real-time PCR and digital PCR assays.

When quantifying the bacterial target DNA in the serially diluted standards of *P. gingivalis*, *A. actinomycetemcomitans*, and *F. nucleatum*, the CV% values of the multiplex dPCR assays ranged from 1.3 − 45.0%, while the CV% values of the multiplex qPCR assays ranged from 3.5 − 34.3% (Figure 2, Supplementary Table S6). A statistically significant (*p* = 0.020) difference in the precision rate of both multiplex dPCR assays (median CV% value: 4.5%) and multiplex qPCR assays (median CV% value: 11.5%) was found when analyzing serially diluted standards with an estimated concentration of 1 × 10^5^ to 1 × 10^1^ copies of target DNA per reaction. The accuracy of the multiplex dPCR assays over the dynamic range was 0.3 − 32.0%, while the accuracy of the multiplex qPCR assays ranged from 0.2 − 18.9% (Supplementary Table S6), with no statistically significant difference between the two assays (*p* = 0.590).

The precision (intra-assay variability) of the multiplex quantitative real-time PCR (qPCR) and digital PCR (dPCR) assays was determined by assessing the coefficient of variation percentage (CV%) in the quantification of bacterial target DNA (in copies per reaction) in serial dilutions of the standards of (a) *Porphyromonas gingivalis*, (b) *Aggregatibacter actinomycetemcomitans*, and (c) *Fusobacterium nucleatum*.

The analytical properties of singleplex dPCR and qPCR assays are shown in Supplementary Table S7 and Supplementary Figure S8.

Testing of human genomic DNA with multiplex and singleplex dPCR assays for periodontal pathobionts did not result in successful DNA amplification (Supplementary Figure S9). All water blanks used to control potential amplicon carryover contaminations were negative for DNA amplification in all dPCR and qPCR runs.

### Diagnostic performance

**Figure 3.**
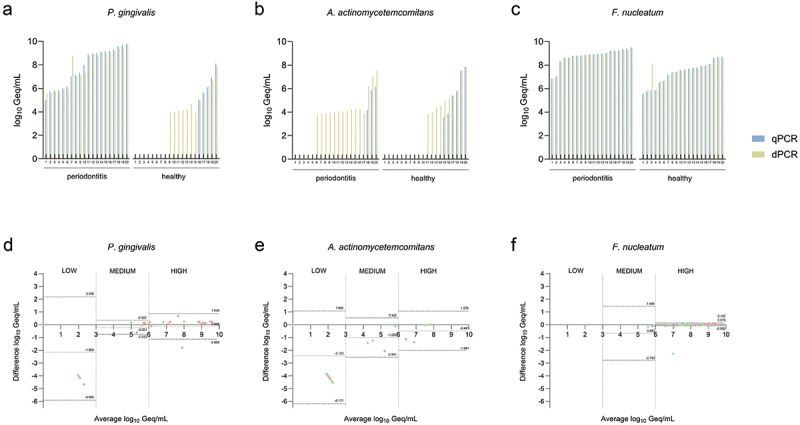
Bacterial loads and Bland-Altman plots for agreement of quantification of *Porphyromonas gingivalis, Aggregatibacter actinomycetemcomitans*, and *Fusobacterium nucleatum* in subgingival plaque samples.

Table 1 shows overall prevalence as well as mean and median counts of targeted bacteria, while raw quantitative data on the targeted bacterial species concentrations can be found in Figure 3(a–c) and Supplementary Table S8. dPCR detected a higher prevalence of *P. gingivalis* and *A. actinomycetemcomitans* compared to qPCR, both in diseased, and even more notably, in healthy periodontium. The difference in bacterial load between the periodontitis and the control group was more pronounced ( > 4.5 log_10_ diff.) for *P. gingivalis*. In contrast, *F. nucleatum* demonstrated an ubiquitous presence in both groups, with similar quantities detected by both methods. The Wilcoxon matched-pairs signed-rank test showed no statistically significant difference between the concentrations of bacterial target DNA that tested positive using dPCR and qPCR in clinical samples (*p* = 0.328). However, when all the samples were considered, including those with qPCR false negatives, a significant difference was observed (*p* = 0.008), highlighting the enhanced sensitivity of dPCR.

Comparison of the multiplex quantitative real-time PCR (qPCR) and digital PCR (dPCR) assays for quantification agreement in subgingival plaque samples. Bacterial load results are subgrouped into categories of periodontitis patients and healthy controls and are ordered in an ascending manner based on qPCR results. (a) Plot comparing the multiplex qPCR and dPCR quantification of *P. gingivalis*, (b) *A. actinomycetemcomitans*, and (c) *F. nucleatum*. The blue-shaded columns represent qPCR data, while the yellow-shaded columns represent dPCR data. The results of the Bland-Altman analysis are divided into arbitrary categories of low ( < 3 log_10_), medium (3 − 6 log_10_), and high ( > 6 log_10_ Geq/mL) bacterial load. The dotted lines indicate the bias, while the dashed lines indicate the limits of agreement (LoA).) Bland-Altman plot comparing the agreement of multiplex qPCR and dPCR assays in the quantification of (d) *P. gingivalis*, (e) *A. actinomycetemcomitans*, and (f) *F. nucleatum*. Red-shaded dots represent samples from periodontitis patients, while green-shaded dots represent samples from healthy periodontium controls. Geq – Genomic equivalents; Periodontitis – periodontitis patient group; Healthy – control patient group; Difference log_10_ Geq/mL— (qPCR-dPCR); Average log_10_ Geq/mL —$\left(\frac{\mathrm{qPCR}+\mathrm{dPCR}}{2}\right)$.

**Table 1.** Frequency of detection, mean and medians of subgingival plaque samples for *Porphyromonas gingivalis*, *Aggregatibacter actinomycetemcomitans*, and *Fusobacterium nucleatum* by the multiplex quantitative real-time PCR (qPCR) and digital PCR (dPCR) assays.

		qPCR	dPCR
*Pg*	Overall prevalence, n (%)	25 (62.50)	32 (80)
	Mean, log_10_ Geq/mL (SD)	4.72 (3.91)	5.41 (3.26)
	Median, log_10_ Geq/mL (IQR)	5.83 (8.73)	5.71 (4.78)
	Periodontitis prevalence, n (%)	20 (100)	20 (100)
	Mean, log_10_ Geq/mL (SD)	7.84 (1.16)	7.80 (1.55)
	Median, log_10_ Geq/mL (IQR)	8.46 (3.15)	8.73 (3.14)
	Healthy prevalence, n (%)	5 (25)	12 (60)
	Mean, log_10_ Geq/mL (SD)	1.59 (2.88)	3.02 (2.72)
	Median, log_10_ Geq/mL (IQR)	0.00 (3.81)	3.99 (4.86)
*Aa*	Overall prevalence, n (%)	9 (22.5)	25 (62.5)
	Mean, log_10_ Geq/mL (SD)	1.26 (2.46)	3.07 (2.62)
	Median, log_10_ Geq/mL (IQR)	0.00 (7.86)	3.94 (3.90)
	Periodontitis prevalence, n (%)	3 (15)	15 (75)
	Mean, log_10_ Geq/mL (SD)	0.81 (2.02)	3.02 (2.72)
	Median, log_10_ Geq/mL (IQR)	0.00 (0.00)	3.97 (3.28)
	Healthy prevalence, n (%)	6 (30)	10 (50)
	Mean log_10_ Geq/mL (SD)	1.70 (2.81)	2.68 (2.91)
	Median log_10_ Geq/mL (IQR)	0.00 (3.82)	1.92 (5.11)
*Fn*	Overall prevalence, n (%)	40 (100)	40 (100)
	Mean, log_10_ Geq/mL (SD)	8.05 (1.12)	8.10 (1.01)
	Median, log_10_ Geq/mL (IQR)	8.48 (1.58)	8.43 (1.41)
	Periodontitis prevalence, n (%)	20 (100)	20 (100)
	Mean, log_10_ Geq/mL (SD)	8.78 (0.70)	8.72 (0.66)
	Median, log_10_ Geq/mL (IQR)	8.93 (0.55)	8.86 (0.49)
	Healthy prevalence, n (%)	20 (100)	20 (100)
	Mean, log_10_ Geq/mL (SD)	7.33 (1.00)	7.47 (0.89)
	Median, log_10_ Geq/mL (IQR)	7.60 (1.44)	7.63 (1.23)

*Pg*—*Porphyromonas gingivalis*; *Aa*—*Aggregatibacter actinomycetemcomitans*; *Fn*—*Fusobacterium nucleatum;* Geq – genome equivalents; SD – standard deviation; IQR – interquartile range; Periodontitis – periodontitis patient group; Healthy – control patient group.

Furthermore, McNemars’s test of the frequency distribution between dPCR and qPCR showed significant differences in the detection of *P. gingivalis* and *A. actinomycetemcomitans* (*p* < 0.001). No variability in detection was observed for *F. nucleatum* since it was omnipresent in both study groups. Following the McNemars’s test, a *post-hoc* power analysis comparing dPCR and qPCR measurements was performed, whereby the frequency of detected bacteria (*n* = 40, and α = 0.05) was selected as the primary outcome. It resulted in a power of 76% and 99% for *P. gingivalis* and *A. actinomycetemcomitans*, respectively. Since the inter-method agreement was perfect for *F. nucleatum* detection, no *post-hoc* power analysis was applicable.

Diagnostic parameters are presented in Table 2 and Supplementary Table S9. The comparison between qPCR and dPCR revealed notable differences in the diagnostic performance for detecting periodontal pathobionts. dPCR consistently demonstrated superior sensitivity, particularly for *P. gingivalis* and *A. actinomycetemcomitans*, indicating its effectiveness in identifying these pathobionts even at lower bacterial loads. However, the lower specificity of dPCR suggested a higher likelihood of false positives, possibly due to the inadequacy of qPCR as a reference standard in the low bacterial load ranges. Despite this, substantial concordance was observed for *P. gingivalis*, while detection discrepancies for *A. actinomycetemcomitans* resulted in poor agreement. In contrast, the omnipresence of *F. nucleatum* in our study population and its relatively high bacterial loads resulted in a high concordance coefficient between the methods.

**Table 2.** Sensitivity and specificity parameters for *Porphyromonas gingivalis*, *Aggregatibacter actinomycetemcomitans*, and *Fusobacterium nucleatum* using the multiplex quantitative real-time PCR (qPCR) assay or the multiplex digital PCR (dPCR) assay as the reference method.

	Reference method	qPCR		dPCR	
		Value	95% CI	Value	95% CI
*Pg*	Sensitivity	0.78	0.61 − 0.89	1.00	0.87 − 1.00
	Specificity	1.00	0.68 − 1.00	0.23	0.12 − 0.39
	Positive Predictive Value	1.00	0.87 − 1.00	0.48	0.35 − 0.61
	Negative Predictive Value	0.53	0.30 − 0.75	1.00	0.68 − 1.00
	Concordance coefficient	0.88	0.87 − 0.93		
*Aa*	Sensitivity	0.36	0.20 − 0.55	1.00	0.70 − 1.00
	Specificity	1.00	0.80 − 1.00	0.48	0.32 − 0.65
	Positive Predictive Value	1.00	0.70 − 1.00	0.36	0.20 − 0.55
	Negative Predictive Value	0.48	0.32 − 0.65	1.00	0.80 − 1.00
	Concordance coefficient	0.56	0.37 − 0.71		
*Fn*	Sensitivity	1.00	0.84 − 1.00	1.00	0.84 − 1.00
	Specificity				
	Positive Predictive Value	1.00	0.84 − 1.00	1.00	0.84 − 1.00
	Negative Predictive Value				
	Concordance coefficient	0.94	0.89 − 0.97		

Pg – Porphyromonas gingivalis; Aa – Aggregatibacter actinomycetemcomitans; Fn – Fusobacterium nucleatum; 95% CI – confidence interval of 95%.

### Bland-Altman analysis

To assess the agreement between qPCR and dPCR quantification in clinical samples, the results were categorized into arbitrary categories of low ( < 3 log_10_), medium (3 − 6 log_10_), and high ( > 6 log_10_ Geq/mL) bacterial load for a more distinct analysis. The analysis revealed a wide range of quantification biases across different pathobionts (Supplementary Table S10). The Bland-Altman analysis (Figure 3(d–f)) indicated good agreement between qPCR and dPCR for medium and high bacterial loads of *P. gingivalis*. For *A. actinomycetemcomitans*, medium and high bacterial loads demonstrated fair to strong agreement between the methods. However, detection discrepancies in the low bacterial load group resulted in a notable bias. Similarly, medium and high bacterial loads of *F. nucleatum* also showed high agreement, with minimal bias. No analysis was performed for the low bacterial load group, as no clinical sample was within this category.

## Discussion

The present study identified substantial differences between dPCR and qPCR performance. dPCR yielded higher detection rates, particularly at low target concentrations in complex subgingival plaque samples, highlighting its superior sensitivity in identifying periodontal pathobionts across various clinical conditions and a broad range of bacterial burden.

dPCR is currently less widespread and more expensive than qPCR but enables the absolute quantification of target nucleic acids based on the Poisson distribution with a high degree of precision without the use of standards and generation of calibration curves [10,28]. The analytical validation of novel multiplex dPCR and qPCR assays showed that the dynamic range for the detection of bacterial target DNA by dPCR measured up to four orders of magnitude due to the positive saturation of the fluorescence signal at the highest measured DNA concentration (i.e. 10^6^ cp/reaction), while the dynamic range of qPCR was much higher – up to nine orders of magnitude [8,29]. The comparison of intra-assay variability confirmed that dPCR has significantly lower CV% values and a higher precision rate; the precision of both methods generally decreases with target DNA concentration, as previously described [29,30]. With the exception of the quantification of standards of *P. gingivalis* and *A. actinomycetemcomitans* (with an estimated input concentration of 1 × 10^1^ cp/reaction), as well as *F. nucleatum* (with an estimated input concentration of 1 × 10^2^ cp/reaction), the CV% and accuracy of both the multiplex dPCR and qPCR assays was found to be within the dynamic range that is predominantly below the threshold for acceptance of quantitative methods (≤25%) [27].

A potential challenge was identified in the diagnostic performance of multiplex dPCR due to its lower dynamic range for the detection of target DNA. When multiple bacterial species are present at very different concentrations in the same clinical sample, targets with high concentrations need to be diluted for accurate quantification, while targets with low concentrations can be tested directly. Nevertheless, no statistically significant differences were observed between the determination of bacterial target DNA concentrations in clinical samples by absolute quantification with dPCR and relative quantification with qPCR, suggesting that appropriate dilution preparation does not substantially affect the reliability of dPCR performance. The Bland-Altman analysis revealed that dPCR and qPCR quantification generally agreed well at medium and high bacterial loads in complex subgingival clinical samples but showed significant discrepancies at lower loads where standard culture detection methods [31,32] and qPCR tend to yield more false negative results. Notably, inconsistencies were observed at lower concentrations of *P. gingivalis* and *A. actinomycetemcomitans*, in both healthy and diseased periodontium. The reason why dPCR detected low concentration targets in a larger number of clinical samples than qPCR may be attributed to the fact that dPCR is less susceptible to competition between targets for PCR reagents due to reaction partitioning and endpoint amplification; it can therefore detect small amounts of a specific target even in the presence of a high background of another target [9,30]. Nevertheless, the clinical implications of detecting these pathogens at such low concentrations remain unclear. Such findings may serve as early indicators for the onset of the disease. However, these results should be interpreted with caution as our study did not distinguish between bacterial strains that might differ in their role in primary pathogenesis.

Despite a small cohort size, the high prevalence of *A. actinomycetemcomitans* detected by dPCR exceed previously reported European epidemiological data [33–39]. This raises questions regarding earlier association studies and the detection of periodontally active sites, and from another perspective, the clinical justification for empirical antibiotic prescriptions based on the mere presence of *A. actinomycetemcomitans* [40]. However, the genotyping of *A. actinomycetemcomitans*, which is a crucial factor in determining the clinical relevance of the phenotype [41,42], was not evaluated in this study.

On the other hand, the traditionally high prevalence of *P. gingivalis* in periodontitis patients [43] remained consistent and high across classical culture [44] and both molecular methods.

In concordance with microbiome studies that identified *F. nucleatum* as part of the core microbiota present in both periodontitis and healthy periodontium [45], we also confirmed the ubiquity of this bacteria. It is worth emphasizing that certain subspecies of *F. nucleatum* are associated with distinct ecological niches and pathologies [46,47]. However, this distinction could not be classified using 16S rRNA-based primers.

Furthermore, there is evidence that these periodontal pathobionts are associated not only with localized periodontal disease but also with systemic diseases, including Alzheimer’s disease and colorectal cancer [48], via defined, bacteria-dependent mechanisms [49,50] that extend beyond the scope of periodontal disease and its status per se. This emphasizes the importance of validated molecular methods capable of reliably detecting targets with low levels of oral bacteria in potentially qPCR-limiting environments [9]. From another perspective, certain dPCR systems allow for the recovery of individual partitions, enabling downstream sequencing, which is an added capability of particular value in both basic and clinical research [9]. A general limitation of PCR-based methods is that they detect both viable and non-viable bacteria, whereas culture-based methods identify only live bacteria. To address this, propidium monoazide (PMA) [51–53] may be used to differentially detect DNA from viable bacteria only, improving the result reliability.

The present study was designed as a proof-of-concept, focused only on three important periodontal pathobionts, and the small sample size limits the generalizability of the findings. Further studies with larger samples, an expanded panel of pathobionts and additional representative oral habitats are therefore needed to confirm these results for different clinical conditions.

## Conclusion

Although qPCR is still considered the gold standard in microbial diagnostics due to its cost-effectiveness and accessibility, it has significant limitations in its ability to detect low bacterial loads within a high background of another target. This can lead to false-negative results and an underestimation of pathogen prevalence. In contrast, dPCR offers quantification without the need for standard curves, higher precision and better performance in detecting low-abundance targets. Although its higher cost and lower availability may limit its routine use, a preliminary cost-benefit analysis suggests that dPCR may be particularly valuable in early disease stages or subclinical conditions, as well as in research where accurate detection of microbial load at low concentrations is critical. Wider clinical application will depend on future studies confirming diagnostic efficacy and economic feasibility.

To the best of our knowledge, this is the first study to use dPCR for the detection and quantification of periodontal pathobionts in clinical periodontal samples. Within the limitations of this study, we can conclude that dPCR is a reliable method for the quantification of periodontal pathobionts in patients with different clinical conditions, including a wide range of bacterial loads. Thanks to its superior sensitivity and precision, it is particularly effective in detecting low bacterial loads, providing more accurate epidemiological data and represents a valuable tool for more accurate microbial diagnostics in periodontal diseases.

## Supplementary Material

R1_Munjakovic_Supplementary Appendix_Final.docx
