# Supplementary material for: Digital PCR outperforms quantitative real-time PCR for the detection and quantification of major periodontal pathobionts
Source: J Oral Microbiol. 2025 Jul 23;17(1):2537439. doi: 10.1080/20002297.2025.2537439 (PMC12288185; doi:10.1080/20002297.2025.2537439)
Supplement: R1_Munjakovic_Supplementary Appendix_Final.docx [file ZJOM_A_2537439_SM8070.docx]

**SUPPLEMENTARY APPENDIX**

Digital PCR outperforms quantitative real-time PCR for the detection and quantification of major periodontal pathobionts

Haris Munjaković^1,2^ *, Katja Povšič^1,2^ *, Mario Poljak^3^, Katja Seme^3^, Rok Gašperšič^1,2^, Lucijan Skubic^3,4^

^1^ Department of Oral Medicine and Periodontology, University Medical Centre Ljubljana, Ljubljana, Slovenia

^2^ Department of Oral Medicine and Periodontology, Faculty of Medicine, University of Ljubljana, Ljubljana, Slovenia

^3^ Institute of Microbiology and Immunology, Faculty of Medicine, University of Ljubljana, Ljubljana, Slovenia

^4^ Clinical Institute for Special Laboratory Diagnostics, University Medical Centre Ljubljana, Ljubljana, Slovenia

*Authors contributing equally to this article.

Address correspondence to Lucijan Skubic; lucijan.skubic@kclj.si

**Bacterial reference strains**

The reference strains *Porphyromonas gingivalis* ATCC 33277 and *Fusobacterium nucleatum* subsp. *polymorphum* DSM 20482 were cultured on blood agar plates (Oxoid No. 2 Blood Agar, Oxoid, Basingstoke, UK) supplemented with 5% sterile horse blood (Biognost, Zagreb, Croatia), 5 mg/L haemin (Sigma-Aldrich, Burlington, Massachusetts, USA), and 1 mg/L menadione (Sigma-Aldrich) under anaerobic conditions and, *Aggregatibacter actinomycetemcomitans* ATCC 43718 under microaerophilic conditions (5% CO₂) at 37°C for 72 hours. After incubation, cells were collected in 1 mL of 0.9% NaCl, harvested by centrifugation at 6000 × g for 10 min, and the pellets were stored at -20°C until DNA extraction.

**DNA extraction**

After defrosting, the samples were vigorously mixed for 30 s and sonicated for 1 min at 40 kHz (Bandelin, Berlin, Germany) to disperse aggregated bacteria. Each sample was then centrifuged at 6000 × g for 10 min, and the supernatants were discarded. DNA was extracted from the pelleted bacterial cells using the QIAamp DNA Mini kit (Qiagen, Hilden, Germany) following the manufacturer's instructions. The extracted DNA was eluted in 200 µL of Milli-Q water (Merck, Darmstadt, Germany) and stored at -20°C until further analysis.

**Primers and probes**

Oligonucleotide primers and double-quenched hydrolysis probes targeting *P. gingivalis, A. actinomycetemcomitans*, and *F.* *nucleatum* (Supplementary Table 2) were designed based on multiple sequence alignments of the 16S rRNA gene nucleotide sequences of different bacterial strains using the MAFFT v7.505 algorithm ^1^. The 16S rRNA gene nucleotide sequences used in the multiple sequence alignments were obtained from the GenBank database (NCBI, 2024), whereas their accession numbers are available in Supplementary Table 3. Specific primer-probe sets were subsequently generated using the Primer3 v0.4.0 web-based application ^2,3^, while their chemical and thermodynamic properties were evaluated using the Net Primer web-based application (PREMIER Biosoft, 2024). The primer-probe sets were synthesized and supplied by Integrated DNA Technologies (Integrated DNA Technologies BVBA—IDT, Leuven, Belgium).

**Quantitative real-time PCR (qPCR) assay**

Multiplex qPCR assay targeting *P. gingivalis*, *A. actinomycetemcomitans* and *F. nucleatum* was performed using the LightCycler 480 Probes Master kit (Roche Diagnostics, Mannheim, Germany) in 20 µL reaction mixtures, each consisting of 5 μL of sample DNA, 10 μL 2× LightCycler 480 Probes Master, 0.4 μM of each of the specific primers, 0.2 μM of each of the specific probes (Supplementary Table 2), and nuclease-free water. The qPCR amplifications were performed using a QuantStudio 7 Pro instrument (Applied Biosystems, Life Technologies, Carlsbad, CA, USA) under the following conditions: initial DNA denaturation for 10 min at 95°C (temperature transition rate of 1.6°C/s), followed by 45 amplification cycles of 10 s at 95°C (1.6°C/s), 30 s at 58°C (1.6°C/s), and 1 s at 72°C (1.6°C/s). Acquisition of the fluorescence signal was performed on specific fluorescence detection channels in a single mode at the end of the elongation step of each amplification cycle. The final step consisted of cooling the reaction mixture to 40°C with a 30 s hold (1.6°C/s). Data were analyzed using the Design & Analysis Software v2.6.0 (Applied Biosystems). The concentration of specific bacterial DNA was determined by the standard curve method, testing 10-fold serially diluted standards of bacterial target DNA, corresponding to 1 × 10^6^ to 1 × 10^−1^ copies of target DNA per reaction. A reaction mixture with nuclease-free water instead of the DNA template (no template control) was used in all qPCR runs as a reference control for potential amplicon carryover contamination.

**Supplementary Table 1.** Inclusion and exclusion criteria for the study population.

| **Inclusion criteria** | **Exclusion criteria** |
| --- | --- |
| - Age 20-80 years - Systemically healthy - No regular systemic medication - Presence of at least 6 teeth in each jaw quadrant | - Regular use of a mouthwash as part of oral hygiene routine - Antibiotic treatment in the last 6 months - Pregnancy - Lactation - Fixed/removable prosthetic devices - Dental implants - Systemic illness (i.e. HIV/AIDS, diabetes mellitus, cancer, bone metabolism diseases, diseases affecting wound healing) - Treatment with immunosuppressive therapy, chemotherapy, radiation therapy, calcium antagonists, antiepileptics, non-steroidal anti-inflammatory drugs |

**Supplementary Table 2.** Primers and double-quenched probes targeting the 16S rRNA gene of *Porphyromonas gingivalis*, *Aggregatibacter actinomycetemcomitans*, and *Fusobacterium nucleatum.*

| Primer/probe | Target | Amplicon Size | Nucleotide Sequence (5′–3′) |
| --- | --- | --- | --- |
| *Pg*_Forward | *Pg* 16S rRNA gene | 194-bp | CGTAAGAATAAGCATCGGCTAACT |
| *Pg*_Reverse |  |  | CACGAATTCCGCCTGCC |
| *Pg*_Probe |  |  | HEX-TCGGTAAGT-ZEN-CAGCGGTGAAACCTGA-IABkFQ |
| *Aa*_Forward | *Aa* 16S rRNA gene | 237-bp | CGGTGTCGATTTGGGGATT |
| *Aa*_Reverse^a^ |  |  | TGCAGCACCTGTCTCAAAGC |
| *Aa*_Probe |  |  | FAM-AGAACTCAG-ZEN-AGATGGGTTTGTGCCTTAGG-IABkFQ |
| *Fn*_Forward | *Fn* 16S rRNA gene | 168-bp | GATTTATTGGGCGTAAAGCG |
| *Fn*_Reverse |  |  | CCCATCGGCATTCCTACAA |
| *Fn*_Probe |  |  | Cy5-AGGGCTCAA-TAO-CTCTGTATTGCGTTGGA -IAbRQSp |

*Pg*—*Porphyromonas gingivalis*; *Aa*—*Aggregatibacter actinomycetemcomitans*; *Fn*—*Fusobacterium nucleatum*; rRNA—ribosomal RNA; bp—base pairs; ^a^ oligonucleotide primer was developed by ^4^.

**Supplementary Table 3.** GenBank accession numbers for the 16S rRNA gene nucleotide sequences of different bacterial strains of *Porphyromonas gingivalis*, *Aggregatibacter actinomycetemcomitans*, and *Fusobacterium nucleatum* used in the multiple sequence alignments in the development of oligonucleotide primers and hydrolysis probes.

*Porphyromonas gingivalis*

| GenBank Accession ID | Name |
| --- | --- |
| PP464148.1 | Porphyromonas gingivalis strain 1 16S ribosomal RNA gene, partial sequence |
| PP464149.1 | Porphyromonas gingivalis strain 2 16S ribosomal RNA gene, partial sequence |
| PP464150.1 | Porphyromonas gingivalis strain 4 16S ribosomal RNA gene, partial sequence |
| PP464150.1 | Porphyromonas gingivalis strain 4 16S ribosomal RNA gene, partial sequence |
| PP464152.1 | Porphyromonas gingivalis strain 14 16S ribosomal RNA gene, partial sequence |
| OR206419.1 | Porphyromonas gingivalis strain LyEC01 16S ribosomal RNA gene, partial sequence |
| OP456623.1 | Porphyromonas gingivalis strain DS2 16S ribosomal RNA gene, partial sequence |
| LC753122.1 | Porphyromonas gingivalis B091 gene for 16S rRNA, partial sequence |
| LC753123.1 | Porphyromonas gingivalis B128 gene for 16S rRNA, partial sequence |
| X73964.1 | P.gingivalis gene for 16S rRNA |
| AF285870.1 | Porphyromonas gingivalis 16S ribosomal RNA gene, partial sequence |
| AB035459.1 | Porphyromonas gingivalis gene for 16S rRNA, partial sequence, strain:ATCC33277 |
| AF414809.1 | Porphyromonas gingivalis strain ATCC 33277 16S ribosomal RNA gene, partial sequence |
| AB035456.1 | Porphyromonas gingivalis gene for 16S rRNA, partial sequence, strain:W83 |
| AB035455.1 | Porphyromonas gingivalis gene for 16S rRNA, partial sequence, strain:FDC 381 |
| AB035457.1 | Porphyromonas gingivalis gene for 16S rRNA, partial sequence, strain:SUNY1021 |
| AB035458.1 | Porphyromonas gingivalis gene for 16S rRNA, partial sequence, strain:A7A1-28 |
| AF287987.1 | Porphyromonas gingivalis 16S ribosomal RNA gene, partial sequence |
| AB910743.1 | Porphyromonas gingivalis gene for 16S ribosomal RNA, partial sequence |
| NR_113086.1 | Porphyromonas gingivalis strain JCM 12257 16S ribosomal RNA, partial sequence |
| PP464152.1 | Porphyromonas gingivalis strain 14 16S ribosomal RNA gene, partial sequence |
| PP464151.1 | Porphyromonas gingivalis strain 7 16S ribosomal RNA gene, partial sequence |
| PP464150.1 | Porphyromonas gingivalis strain 4 16S ribosomal RNA gene, partial sequence |
| PP464148.1 | Porphyromonas gingivalis strain 1 16S ribosomal RNA gene, partial sequence |
| PP464149.1 | Porphyromonas gingivalis strain 2 16S ribosomal RNA gene, partial sequence |
| OR206419.1 | Porphyromonas gingivalis strain LyEC01 16S ribosomal RNA gene, partial sequence |
| MN064795.1 | Porphyromonas gingivalis strain ANA230 16S ribosomal RNA gene, partial sequence |
| KX108929.1 | Porphyromonas gingivalis strain IR-TUMS/BPG5 16S ribosomal RNA gene, partial sequence |
| KT222964.1 | Porphyromonas gingivalis strain CP52 16S ribosomal RNA gene, partial sequence |
| KT191027.1 | Porphyromonas gingivalis strain CP51 16S ribosomal RNA gene, partial sequence |
| KR074428.1 | Porphyromonas gingivalis strain CP44 16S ribosomal RNA gene, partial sequence |
| KR074427.1 | Porphyromonas gingivalis strain CP41 16S ribosomal RNA gene, partial sequence |
| KR074426.1 | Porphyromonas gingivalis strain CP40 16S ribosomal RNA gene, partial sequence |
| KR074425.1 | Porphyromonas gingivalis strain CP38 16S ribosomal RNA gene, partial sequence |
| KR074424.1 | Porphyromonas gingivalis strain CP33 16S ribosomal RNA gene, partial sequence |
| KR074423.1 | Porphyromonas gingivalis strain CP27 16S ribosomal RNA gene, partial sequence |
| KR074422.1 | Porphyromonas gingivalis strain CP24 16S ribosomal RNA gene, partial sequence |
| KR351312.1 | Porphyromonas gingivalis strain 381 16S ribosomal RNA gene, partial sequence |
| KM897673.1 | Porphyromonas gingivalis strain CP4 16S ribosomal RNA gene, partial sequence |
| AF414820.1 | Porphyromonas gingivalis strain 30-PGI 16S ribosomal RNA gene, partial sequence |
| AF414819.1 | Porphyromonas gingivalis strain 29-PGI 16S ribosomal RNA gene, partial sequence |
| AF414818.1 | Porphyromonas gingivalis strain 28-PGI 16S ribosomal RNA gene, partial sequence |
| AF414817.1 | Porphyromonas gingivalis strain 27-PGI 16S ribosomal RNA gene, partial sequence |
| AF414816.1 | Porphyromonas gingivalis strain 26-PGI 16S ribosomal RNA gene, partial sequence |
| AF414815.1 | Porphyromonas gingivalis strain 25-PGI 16S ribosomal RNA gene, partial sequence |
| AF414814.1 | Porphyromonas gingivalis strain 42-PGI 16S ribosomal RNA gene, partial sequence |
| AF414813.1 | Porphyromonas gingivalis strain 40-PGI 16S ribosomal RNA gene, partial sequence |
| AF414812.1 | Porphyromonas gingivalis strain 3-PGI 16S ribosomal RNA gene, partial sequence |
| AF414811.1 | Porphyromonas gingivalis strain 2-PGI 16S ribosomal RNA gene, partial sequence |
| AF414810.1 | Porphyromonas gingivalis strain 1-PGI 16S ribosomal RNA gene, partial sequence |
| OR206493.1 | Porphyromonas gingivalis strain LyG-2 16S ribosomal RNA gene, partial sequence |
| OR206492.1 | Porphyromonas gingivalis strain LyG-1 16S ribosomal RNA gene, partial sequence |
| OP456623.1 | Porphyromonas gingivalis strain DS2 16S ribosomal RNA gene, partial sequence |
| MT434761.1 | Porphyromonas gingivalis strain KCOM 3188 16S ribosomal RNA gene, partial sequence |
| MT433917.1 | Porphyromonas gingivalis strain KCOM 3190 (=JS297) 16S ribosomal RNA gene, partial sequence |
| MT323063.1 | Porphyromonas gingivalis strain KCOM 3153 (=JS260) 16S ribosomal RNA gene, partial sequence |
| MT312846.1 | Porphyromonas gingivalis strain KCOM 3131 (=JS238) 16S ribosomal RNA gene, partial sequence |
| MT256291.1 | Porphyromonas gingivalis strain KCOM 3001 (=ChDC KB54) 16S ribosomal RNA gene, partial sequence |
| MN044791.1 | Porphyromonas gingivalis strain P.gingivalis6049 16S ribosomal RNA gene, partial sequence |
| MN044790.1 | Porphyromonas gingivalis strain P.gingivalis2381 16S ribosomal RNA gene, partial sequence |
| KU569560.1 | Porphyromonas gingivalis strain W50 16S ribosomal RNA gene, partial sequence; 16S-23S ribosomal RNA intergenic spacer, complete sequence; and 23S ribosomal RNA gene, partial sequence |
| KF933759.1 | Porphyromonas gingivalis strain JS3 16S ribosomal RNA gene, partial sequence |
| KF933758.1 | Porphyromonas gingivalis strain JS2 16S ribosomal RNA gene, partial sequence |
| KF933757.1 | Porphyromonas gingivalis strain JS1 16S ribosomal RNA gene, partial sequence |
| AB910743.1 | Porphyromonas gingivalis gene for 16S ribosomal RNA, partial sequence |
| AB547661.1 | Porphyromonas gingivalis gene for 16S ribosomal RNA, partial sequence, strain: JCM 8525 |
| JQ894505.1 | Porphyromonas gingivalis strain AS2 16S ribosomal RNA gene, partial sequence |
| AB547660.1 | Porphyromonas gingivalis gene for 16S ribosomal RNA, partial sequence, strain: JCM 12257 |
| GU418126.1 | Porphyromonas gingivalis clone WWP_SS9_G31 16S ribosomal RNA gene, partial sequence |
| GU418125.1 | Porphyromonas gingivalis clone WWP_SS8_P09 16S ribosomal RNA gene, partial sequence |
| GU418124.1 | Porphyromonas gingivalis clone WWP_SS7_P30 16S ribosomal RNA gene, partial sequence |
| GU418123.1 | Porphyromonas gingivalis clone WWP_SS3_P17 16S ribosomal RNA gene, partial sequence |
| GU418122.1 | Porphyromonas gingivalis clone WWP_SS2_G56 16S ribosomal RNA gene, partial sequence |
| GU418067.1 | Porphyromonas gingivalis clone WWP_SS8_P29 16S ribosomal RNA gene, partial sequence |
| GU418065.1 | Porphyromonas gingivalis clone WWP_SS8_P17 16S ribosomal RNA gene, partial sequence |
| GU418066.1 | Porphyromonas gingivalis clone WWP_SS8_P28 16S ribosomal RNA gene, partial sequence |
| GU418064.1 | Porphyromonas gingivalis clone WWP_SS8_P14 16S ribosomal RNA gene, partial sequence |
| GU418063.1 | Porphyromonas gingivalis clone WWP_SS8_P03 16S ribosomal RNA gene, partial sequence |
| GU418062.1 | Porphyromonas gingivalis clone WWP_SS8_P02 16S ribosomal RNA gene, partial sequence |
| GU418061.1 | Porphyromonas gingivalis clone WWP_SS7_G23 16S ribosomal RNA gene, partial sequence |
| GU418060.1 | Porphyromonas gingivalis clone WWP_SS7_G18 16S ribosomal RNA gene, partial sequence |
| GU418059.1 | Porphyromonas gingivalis clone WWP_SS6_P19 16S ribosomal RNA gene, partial sequence |
| GU418058.1 | Porphyromonas gingivalis clone WWP_SS6_C31 16S ribosomal RNA gene, partial sequence |
| GU418057.1 | Porphyromonas gingivalis clone WWP_SS6_C28 16S ribosomal RNA gene, partial sequence |
| GU418056.1 | Porphyromonas gingivalis clone WWP_SS6_C21 16S ribosomal RNA gene, partial sequence |
| GU418055.1 | Porphyromonas gingivalis clone WWP_SS6_C09 16S ribosomal RNA gene, partial sequence |
| GU418054.1 | Porphyromonas gingivalis clone WWP_SS2_G03 16S ribosomal RNA gene, partial sequence |
| GU418053.1 | Porphyromonas gingivalis clone WWP_SS2_C43 16S ribosomal RNA gene, partial sequence |
| GU418052.1 | Porphyromonas gingivalis clone WWP_SS10_G03 16S ribosomal RNA gene, partial sequence |
| GU418051.1 | Porphyromonas gingivalis clone WWP_SS1_P73 16S ribosomal RNA gene, partial sequence |
| L16492.1 | Porphyromonas gingivalis ATCC 33277 16S ribosomal RNA gene, complete sequence |

*Aggregatibacter actinomycetemcomitans*

| GenBank Accession ID | Name |
| --- | --- |
| FN547995.1 | Aggregatibacter actinomycetemcomitans partial 16S rRNA gene, strain HG2748/103284, serovar E' |
| FN547994.1 | Aggregatibacter actinomycetemcomitans partial 16S rRNA gene, strain HG2590/101894, serovar E' |
| FN547993.1 | Aggregatibacter actinomycetemcomitans partial 16S rRNA gene, strain HG2542/101672, serovar E' |
| FN547992.1 | Aggregatibacter actinomycetemcomitans partial 16S rRNA gene, strain HG2029/132139, serovar E' |
| FN547991.1 | Aggregatibacter actinomycetemcomitans partial 16S rRNA gene, strain HG2856/104011, serovar E' |
| FN547990.1 | Aggregatibacter actinomycetemcomitans partial 16S rRNA gene, strain HG2514/101373, serovar E' |
| FN547989.1 | Aggregatibacter actinomycetemcomitans partial 16S rRNA gene, strain HG3807/37.1, serovar E |
| FN547988.1 | Aggregatibacter actinomycetemcomitans partial 16S rRNA gene, strain HG3903/204.1, serovar E' |
| FN547987.1 | Aggregatibacter actinomycetemcomitans partial 16S rRNA gene, strain HG3902/158.2, serovar E' |
| FN547986.1 | Aggregatibacter actinomycetemcomitans partial 16S rRNA gene, strain HG3900/149.3-02, serovar E' |
| FN547985.1 | Aggregatibacter actinomycetemcomitans partial 16S rRNA gene, strain HG3020/149.1-94, serovar E' |
| FN547984.1 | Aggregatibacter actinomycetemcomitans partial 16S rRNA gene, strain HG3885/125.4, serovar E |
| FN547983.1 | Aggregatibacter actinomycetemcomitans partial 16S rRNA gene, strain HG3871/119.1, serovar E' |
| FN547982.1 | Aggregatibacter actinomycetemcomitans partial 16S rRNA gene, strain HG3846/100.1, serovar E' |
| FN547981.1 | Aggregatibacter actinomycetemcomitans partial 16S rRNA gene, strain HG3789/10.1, serovar E' |
| FN547980.1 | Aggregatibacter actinomycetemcomitans partial 16S rRNA gene, strain HG3908/HK961/CCUG 51669, serovar E' |
| FN547979.1 | Aggregatibacter actinomycetemcomitans partial 16S rRNA gene, strain HG3907/HK929/CCUG 51668, serovar E |
| FN547978.1 | Aggregatibacter actinomycetemcomitans partial 16S rRNA gene, strain HG3906/HK443, serovar C |
| FN547977.1 | Aggregatibacter actinomycetemcomitans partial 16S rRNA gene, strain HG3909/HK1002/CCUG 12604, serovar D |
| FN547976.1 | Aggregatibacter actinomycetemcomitans partial 16S rRNA gene, strain HG90/Y4, serovar B |
| FN547975.1 | Aggregatibacter actinomycetemcomitans partial 16S rRNA gene, strain HG683T/NCTC9710T, serovar C |
| FN547974.1 | Aggregatibacter actinomycetemcomitans partial 16S rRNA gene, strain HG1652/OMZ546, serovar E' |
| FN547973.1 | Aggregatibacter actinomycetemcomitans partial 16S rRNA gene, strain HG1651/OMZ541, serovar E' |
| FN547971.1 | Aggregatibacter actinomycetemcomitans partial 16S rRNA gene, strain HG1229/538, serovar B |
| FN547970.1 | Aggregatibacter actinomycetemcomitans partial 16S rRNA gene, strain HG1226/528, serovar C |
| FN547969.1 | Aggregatibacter actinomycetemcomitans partial 16S rRNA gene, strain HG1181/556, serovar A |
| FN547968.1 | Aggregatibacter actinomycetemcomitans partial 16S rRNA gene, strain HG1223/ATCC29523, serovar A |
| FN547967.1 | Aggregatibacter actinomycetemcomitans partial 16S rRNA gene, strain HG2217/3381, serovar D |
| FN547965.1 | Aggregatibacter actinomycetemcomitans partial 16S rRNA gene, strain HG3904/231.4, serovar E' |
| FN547964.1 | Aggregatibacter actinomycetemcomitans partial 16S rRNA gene, strain HG2218/1344, serovar D |
| FN547963.1 | Aggregatibacter actinomycetemcomitans partial 16S rRNA gene, strain HG2864/104084, serovar F |
| LR899004.1 | Aggregatibacter actinomycetemcomitans partial 16S rRNA gene |
| HQ188689.1 | Aggregatibacter actinomycetemcomitans isolate D123 16S ribosomal RNA gene, partial sequence |
| AB512012.1 | Aggregatibacter actinomycetemcomitans gene for 16S ribosomal RNA, partial sequence, strain: IDH781 |
| OQ947776.1 | Aggregatibacter actinomycetemcomitans strain Sdc_ortho14 16S ribosomal RNA gene, partial sequence |
| NR_029171.1 | Aggregatibacter actinomycetemcomitans strain 277/49-50(8) 16S ribosomal RNA, partial sequence |
| AY362885.1 | Aggregatibacter actinomycetemcomitans strain ATCC 29523 16S ribosomal RNA gene, partial sequence |
| LC383929.1 | Aggregatibacter actinomycetemcomitans JCM 2435 gene for 16S ribosomal RNA, partial sequence |
| M75035.1 | Aggregatibacter actinomycetemcomitans strain FDC Y4 16S ribosomal RNA gene, partial sequence |
| M75037.1 | Aggregatibacter actinomycetemcomitans strain ATCC 29524 16S ribosomal RNA gene, partial sequence |

*Fusobacterium nucleatum*

| GenBank Accession ID | Name |
| --- | --- |
| AJ810280.1 | Fusobacterium nucleatum partial 16S rRNA gene, strain OMZ 986 |
| LR880973.1 | Fusobacterium nucleatum partial 16S rRNA gene |
| AJ810278.1 | Fusobacterium nucleatum partial 16S rRNA gene, strain OMZ 985 |
| AJ810277.1 | Fusobacterium nucleatum partial 16S rRNA gene, strain OMZ 274 |
| AJ810275.1 | Fusobacterium nucleatum partial 16S rRNA gene, strain JC-208 |
| AJ810276.1 | Fusobacterium nucleatum partial 16S rRNA gene, strain KP-F2 |
| EU419227.1 | Fusobacterium nucleatum isolate 2-1-50A 16S ribosomal RNA gene, partial sequence |
| EU419226.1 | Fusobacterium nucleatum isolate 3-1-48A 16S ribosomal RNA gene, partial sequence |
| EU419225.1 | Fusobacterium nucleatum isolate 3-1-46B2 16S ribosomal RNA gene, partial sequence |
| EU419224.1 | Fusobacterium nucleatum isolate 3-2-44B 16S ribosomal RNA gene, partial sequence |
| EU419222.1 | Fusobacterium nucleatum isolate 2-A-37 16S ribosomal RNA gene, partial sequence |
| EU419221.1 | Fusobacterium nucleatum isolate 3-1-37BFAA 16S ribosomal RNA gene, partial sequence |
| EU419220.1 | Fusobacterium nucleatum isolate 2-1-37FAA 16S ribosomal RNA gene, partial sequence |
| EU419219.1 | Fusobacterium nucleatum isolate 1-A-36 16S ribosomal RNA gene, partial sequence |
| EU419218.1 | Fusobacterium nucleatum isolate 3-1-36A2 16S ribosomal RNA gene, partial sequence |
| EU419217.1 | Fusobacterium nucleatum isolate 3-1-33 16S ribosomal RNA gene, partial sequence |
| EU419215.1 | Fusobacterium nucleatum isolate 3-1-27 16S ribosomal RNA gene, partial sequence |
| EU419214.1 | Fusobacterium nucleatum isolate 2-A-13 16S ribosomal RNA gene, partial sequence |
| EU419212.1 | Fusobacterium nucleatum isolate 4-1-13 16S ribosomal RNA gene, partial sequence |
| EU419210.1 | Fusobacterium nucleatum isolate 2-A-7 16S ribosomal RNA gene, partial sequence |
| EU419209.1 | Fusobacterium nucleatum isolate 1-A-7 16S ribosomal RNA gene, partial sequence |
| EU419207.1 | Fusobacterium nucleatum isolate 1-A-4D 16S ribosomal RNA gene, partial sequence |
| EU419206.1 | Fusobacterium nucleatum isolate 3-2-4 16S ribosomal RNA gene, partial sequence |
| EU419205.1 | Fusobacterium nucleatum isolate 21-1A 16S ribosomal RNA gene, partial sequence |
| EU419204.1 | Fusobacterium nucleatum isolate 13-3c 16S ribosomal RNA gene, partial sequence |
| EU419203.1 | Fusobacterium nucleatum isolate 11-3-2 16S ribosomal RNA gene, partial sequence |
| EU419202.1 | Fusobacterium nucleatum isolate 7-1 16S ribosomal RNA gene, partial sequence |
| EU419201.1 | Fusobacterium nucleatum isolate 4-8 16S ribosomal RNA gene, partial sequence |
| FJ471660.2 | Fusobacterium nucleatum strain ChDC F318 16S ribosomal RNA gene, partial sequence |
| FJ471648.2 | Fusobacterium nucleatum strain ChDC F218 16S ribosomal RNA gene, partial sequence |
| FJ471645.2 | Fusobacterium nucleatum strain ChDC F175 16S ribosomal RNA gene, partial sequence |
| FJ471639.2 | Fusobacterium nucleatum strain ChDC F113 16S ribosomal RNA gene, partial sequence |
| FJ471638.2 | Fusobacterium nucleatum strain ChDC F37 16S ribosomal RNA gene, partial sequence |
| FJ471664.1 | Fusobacterium nucleatum strain ChDC F332 16S ribosomal RNA gene, partial sequence |
| FJ471663.1 | Fusobacterium nucleatum strain ChDC F330 16S ribosomal RNA gene, partial sequence |
| FJ471662.1 | Fusobacterium nucleatum strain ChDC F324 16S ribosomal RNA gene, partial sequence |
| FJ471661.1 | Fusobacterium nucleatum strain ChDC F319 16S ribosomal RNA gene, partial sequence |
| FJ471659.1 | Fusobacterium nucleatum strain ChDC F317 16S ribosomal RNA gene, partial sequence |
| FJ471658.1 | Fusobacterium nucleatum strain ChDC F316 16S ribosomal RNA gene, partial sequence |
| FJ471657.1 | Fusobacterium nucleatum strain ChDC F315 16S ribosomal RNA gene, partial sequence |
| FJ471656.1 | Fusobacterium nucleatum strain ChDC F313 16S ribosomal RNA gene, partial sequence |
| FJ471655.1 | Fusobacterium nucleatum strain ChDC F311 16S ribosomal RNA gene, partial sequence |
| FJ471654.1 | Fusobacterium nucleatum strain ChDC F310 16S ribosomal RNA gene, partial sequence |
| FJ471653.1 | Fusobacterium nucleatum strain ChDC F309 16S ribosomal RNA gene, partial sequence |
| FJ471652.1 | Fusobacterium nucleatum strain ChDC F306 16S ribosomal RNA gene, partial sequence |
| FJ471651.1 | Fusobacterium nucleatum strain ChDC F305 16S ribosomal RNA gene, partial sequence |
| FJ471649.1 | Fusobacterium nucleatum strain ChDC F290 16S ribosomal RNA gene, partial sequence |
| FJ471646.1 | Fusobacterium nucleatum strain ChDC F186 16S ribosomal RNA gene, partial sequence |
| FJ471642.1 | Fusobacterium nucleatum strain ChDC F130 16S ribosomal RNA gene, partial sequence |
| FJ471640.1 | Fusobacterium nucleatum strain ChDC F119 16S ribosomal RNA gene, partial sequence |
| FJ471637.1 | Fusobacterium nucleatum strain ChDC F8 16S ribosomal RNA gene, partial sequence |
| EF153314.1 | Fusobacterium nucleatum strain Ulm 18 16S ribosomal RNA gene, partial sequence |
| EF153311.1 | Fusobacterium nucleatum strain Ulm 15 16S ribosomal RNA gene, partial sequence |
| KM023647.1 | Fusobacterium nucleatum strain FDC 364 16S ribosomal RNA gene, partial sequence |
| DQ440565.1 | Fusobacterium nucleatum strain Ulm C 16S ribosomal RNA gene, partial sequence |
| DQ440564.1 | Fusobacterium nucleatum strain Ulm B 16S ribosomal RNA gene, partial sequence |
| DQ440563.1 | Fusobacterium nucleatum strain Ulm A 16S ribosomal RNA gene, partial sequence |
| DQ440558.1 | Fusobacterium nucleatum strain Ulm 9 16S ribosomal RNA gene, partial sequence |
| DQ440557.1 | Fusobacterium nucleatum strain Ulm 8 16S ribosomal RNA gene, partial sequence |
| DQ440556.1 | Fusobacterium nucleatum strain Ulm 7 16S ribosomal RNA gene, partial sequence |
| AB514450.1 | Fusobacterium nucleatum gene for 16S ribosomal RNA, partial sequence, strain: IMU102 |
| EF089177.1 | Fusobacterium nucleatum strain Ulm 14 16S ribosomal RNA gene, partial sequence |
| OP474078.1 | Fusobacterium nucleatum strain c22_VSa_1 16S ribosomal RNA gene, partial sequence |
| OP107897.1 | Fusobacterium nucleatum strain JD-Fn6 16S ribosomal RNA gene, partial sequence |
| OP107896.1 | Fusobacterium nucleatum strain JD-Fn3 16S ribosomal RNA gene, partial sequence |
| OP051099.1 | Fusobacterium nucleatum strain JD-Fn8 16S ribosomal RNA gene, partial sequence |
| OP051098.1 | Fusobacterium nucleatum strain JD-Fn7 16S ribosomal RNA gene, partial sequence |
| OP051097.1 | Fusobacterium nucleatum strain JD-Fn5 16S ribosomal RNA gene, partial sequence |
| OP051096.1 | Fusobacterium nucleatum strain JD-Fn4 16S ribosomal RNA gene, partial sequence |
| OP051095.1 | Fusobacterium nucleatum strain JD-Fn2 16S ribosomal RNA gene, partial sequence |
| OP051094.1 | Fusobacterium nucleatum strain JD-Fn1 16S ribosomal RNA gene, partial sequence |
| ON864055.1 | Fusobacterium nucleatum strain SSFN3 16S ribosomal RNA gene, partial sequence |
| ON864054.1 | Fusobacterium nucleatum strain SSFN2 16S ribosomal RNA gene, partial sequence |
| ON864053.1 | Fusobacterium nucleatum strain SSFN1 16S ribosomal RNA gene, partial sequence |
| MZ387993.1 | Fusobacterium nucleatum strain P127p (10) 16S ribosomal RNA gene, partial sequence |
| MW729390.1 | Fusobacterium nucleatum strain KCOM 3973 (=ChDC P045-Pg3) 16S ribosomal RNA gene, partial sequence |
| MW687116.1 | Fusobacterium nucleatum strain KCOM 3933 (=ChDC P038-Pg4) 16S ribosomal RNA gene, partial sequence |
| MW659081.1 | Fusobacterium nucleatum strain KCOM 3838 (=ChDC P035-Pg2) 16S ribosomal RNA gene, partial sequence |
| MW567136.1 | Fusobacterium nucleatum strain c15Ua_6_AN 16S ribosomal RNA gene, partial sequence |
| MW599795.1 | Fusobacterium nucleatum strain KCOM 3820 (=ChDC P033-Pg3) 16S ribosomal RNA gene, partial sequence |
| MW581930.1 | Fusobacterium nucleatum strain KCOM 3799 (=ChDC P031-Pg3) 16S ribosomal RNA gene, partial sequence |
| MW563866.1 | Fusobacterium nucleatum strain KCOM 2855 (= JS36) 16S ribosomal RNA gene, partial sequence |
| MK838562.1 | Fusobacterium nucleatum strain UC01 16S ribosomal RNA gene, partial sequence |
| MN988852.1 | Fusobacterium nucleatum strain GS17A 16S ribosomal RNA gene, partial sequence |
| KX096299.1 | Fusobacterium nucleatum culture KCOM:2598 16S ribosomal RNA gene, partial sequence |
| KX096298.1 | Fusobacterium nucleatum culture KCOM:2597 16S ribosomal RNA gene, partial sequence |
| KX096297.1 | Fusobacterium nucleatum culture KCOM:2596 16S ribosomal RNA gene, partial sequence |
| KX096295.1 | Fusobacterium nucleatum culture KCOM:2594 16S ribosomal RNA gene, partial sequence |
| KX096294.1 | Fusobacterium nucleatum culture KCOM:2591 16S ribosomal RNA gene, partial sequence |
| KX096285.1 | Fusobacterium nucleatum culture KCOM:2582 16S ribosomal RNA gene, partial sequence |
| KX096279.1 | Fusobacterium nucleatum culture KCOM:2576 16S ribosomal RNA gene, partial sequence |
| KX096276.1 | Fusobacterium nucleatum culture KCOM:2573 16S ribosomal RNA gene, partial sequence |
| KX096275.1 | Fusobacterium nucleatum culture KCOM:2572 16S ribosomal RNA gene, partial sequence |
| KX692281.1 | Fusobacterium nucleatum strain RCC01 16S ribosomal RNA gene, partial sequence |
| KU321259.1 | Fusobacterium nucleatum strain RHI 4184 16S ribosomal RNA gene, partial sequence |
| KC999390.1 | Fusobacterium nucleatum 16S ribosomal RNA gene, partial sequence |
| KF444254.2 | Fusobacterium nucleatum strain YWH7388 16S ribosomal RNA gene, partial sequence |
| KF444248.2 | Fusobacterium nucleatum strain YWH7361 16S ribosomal RNA gene, partial sequence |
| KF444266.1 | Fusobacterium nucleatum strain YWH7415 16S ribosomal RNA gene, partial sequence |
| KF444265.1 | Fusobacterium nucleatum strain YWH7414 16S ribosomal RNA gene, partial sequence |
| KF444264.1 | Fusobacterium nucleatum strain YWH7413 16S ribosomal RNA gene, partial sequence |
| KF444263.1 | Fusobacterium nucleatum strain YWH7407 16S ribosomal RNA gene, partial sequence |
| KF444262.1 | Fusobacterium nucleatum strain YWH7402 16S ribosomal RNA gene, partial sequence |
| KF444261.1 | Fusobacterium nucleatum strain YWH7401 16S ribosomal RNA gene, partial sequence |
| KF444260.1 | Fusobacterium nucleatum strain YWH7397 16S ribosomal RNA gene, partial sequence |
| KF444259.1 | Fusobacterium nucleatum strain YWH7396 16S ribosomal RNA gene, partial sequence |
| KF444258.1 | Fusobacterium nucleatum strain YWH7395 16S ribosomal RNA gene, partial sequence |
| KF444257.1 | Fusobacterium nucleatum strain YWH7392 16S ribosomal RNA gene, partial sequence |
| KF444255.1 | Fusobacterium nucleatum strain YWH7389 16S ribosomal RNA gene, partial sequence |
| KF444256.1 | Fusobacterium nucleatum strain YWH7390 16S ribosomal RNA gene, partial sequence |
| KF444252.1 | Fusobacterium nucleatum strain YWH7376 16S ribosomal RNA gene, partial sequence |
| KF444253.1 | Fusobacterium nucleatum strain YWH7387 16S ribosomal RNA gene, partial sequence |
| KF444251.1 | Fusobacterium nucleatum strain YWH7372 16S ribosomal RNA gene, partial sequence |
| KF444250.1 | Fusobacterium nucleatum strain YWH7371 16S ribosomal RNA gene, partial sequence |
| KF444249.1 | Fusobacterium nucleatum strain YWH7370 16S ribosomal RNA gene, partial sequence |
| KF444247.1 | Fusobacterium nucleatum strain YWH7360 16S ribosomal RNA gene, partial sequence |
| KF444246.1 | Fusobacterium nucleatum strain YWH7351 16S ribosomal RNA gene, partial sequence |
| KF444245.1 | Fusobacterium nucleatum strain YWH7349 16S ribosomal RNA gene, partial sequence |
| KF444244.1 | Fusobacterium nucleatum strain YWH7338 16S ribosomal RNA gene, partial sequence |
| KF444243.1 | Fusobacterium nucleatum strain YWH7199 16S ribosomal RNA gene, partial sequence |
| KF444242.1 | Fusobacterium nucleatum strain YWH7091 16S ribosomal RNA gene, partial sequence |
| KF444241.1 | Fusobacterium nucleatum strain YWH7079 16S ribosomal RNA gene, partial sequence |
| KF444240.1 | Fusobacterium nucleatum strain YWH7074 16S ribosomal RNA gene, partial sequence |
| KF444239.1 | Fusobacterium nucleatum strain YWH7073 16S ribosomal RNA gene, partial sequence |
| KF444238.1 | Fusobacterium nucleatum strain YWH7071 16S ribosomal RNA gene, partial sequence |
| KF444237.1 | Fusobacterium nucleatum strain YWH7070 16S ribosomal RNA gene, partial sequence |
| KF444236.1 | Fusobacterium nucleatum strain YWH7069 16S ribosomal RNA gene, partial sequence |
| KF444235.1 | Fusobacterium nucleatum strain YWH7056 16S ribosomal RNA gene, partial sequence |
| KF444234.1 | Fusobacterium nucleatum strain YWH7055 16S ribosomal RNA gene, partial sequence |
| KF444233.1 | Fusobacterium nucleatum strain YWH7054 16S ribosomal RNA gene, partial sequence |
| KF444232.1 | Fusobacterium nucleatum strain YWH7053 16S ribosomal RNA gene, partial sequence |
| KF444231.1 | Fusobacterium nucleatum strain YWH7051 16S ribosomal RNA gene, partial sequence |
| KF444230.1 | Fusobacterium nucleatum strain YWH7049 16S ribosomal RNA gene, partial sequence |
| KF444229.1 | Fusobacterium nucleatum strain YWH7048 16S ribosomal RNA gene, partial sequence |
| KF444228.1 | Fusobacterium nucleatum strain YWH7046 16S ribosomal RNA gene, partial sequence |
| KF444227.1 | Fusobacterium nucleatum strain YWH7045 16S ribosomal RNA gene, partial sequence |
| KF444226.1 | Fusobacterium nucleatum strain YWH7013 16S ribosomal RNA gene, partial sequence |
| KF444225.1 | Fusobacterium nucleatum strain YWH7010 16S ribosomal RNA gene, partial sequence |
| HM347082.1 | Fusobacterium nucleatum strain WAL 9085 16S ribosomal RNA gene, partial sequence |
| HM347081.1 | Fusobacterium nucleatum strain Ac630 16S ribosomal RNA gene, partial sequence |
| HM347079.1 | Fusobacterium nucleatum strain WAL 10126 16S ribosomal RNA gene, partial sequence |
| HM347077.1 | Fusobacterium nucleatum strain RMA 8759 16S ribosomal RNA gene, partial sequence |
| HM347069.1 | Fusobacterium nucleatum strain RMA 8756 16S ribosomal RNA gene, partial sequence |
| HM347067.1 | Fusobacterium nucleatum strain RMA 7176 16S ribosomal RNA gene, partial sequence |
| HM347066.1 | Fusobacterium nucleatum strain RMA 7161 16S ribosomal RNA gene, partial sequence |
| HM347057.1 | Fusobacterium nucleatum strain WAL 9696 16S ribosomal RNA gene, partial sequence |
| GU561358.1 | Fusobacterium nucleatum strain TTO6 16S ribosomal RNA gene, partial sequence |
| GU561357.1 | Fusobacterium nucleatum strain GEJ10 16S ribosomal RNA gene, partial sequence |
| GU561356.1 | Fusobacterium nucleatum strain SG9 16S ribosomal RNA gene, partial sequence |
| GU561355.1 | Fusobacterium nucleatum strain AGU7 16S ribosomal RNA gene, partial sequence |
| AF543300.1 | Fusobacterium nucleatum 16S ribosomal RNA gene, partial sequence |
| GU412556.1 | Fusobacterium nucleatum clone WWP_SS6_P17 16S ribosomal RNA gene, partial sequence |
| GU412555.1 | Fusobacterium nucleatum clone WWP_SS10_P10 16S ribosomal RNA gene, partial sequence |

**Supplementary Table 4.** Demographic and clinical characteristics of the treatment groups at baseline.

|  | Periodontitis group | Healthy group | p-value |
| --- | --- | --- | --- |
| Age (years, mean ± SD) | 48.2 ± 11.3 | 43.3 ± 12.3 | 0.203 |
| Male subjects (n, [%]) | 9 (45) | 6 (30) | 0.333 |
| Smokers (n, [%]) | 4 (20) | 2 (10F) | 0.382 |
| Number of teeth (n, mean ± SD) | 24.3 ± 3.3 | 26.8 ± 2.2 | < 0.001* |
| PD at sampling site (mm, mean ± SD) | 8.2 ± 2.0 | 2.9 ± 0.1 | < 0.001* |
| REC at sampling site (mm, mean ± SD) | 0.6 ± 0.2 | 0.4 ± 0.2 | 0.153 |
| CAL at sampling site (mm, mean ± SD) | 9.6 ± 2.4 | 3.2 ± 0.3 | < 0.001* |
| BOP at sampling site (%, mean ± SD) | 94.3 ± 5.3 | 6.7 ± 2.2 | < 0.001* |
| PlI (%, mean ± SD) | 37.0 ± 21.2 | 7.8 ± 2.0 | < 0.001* |

SD—standard deviation; PlI—plaque index; PD—probing depth; REC—recession; CAL—clinical attachment loss; BOP—bleeding on probing; * – statistical significance at α = 0.05

**Supplementary Table 5.**  Allocation of periodontitis patients based on their stage and grade, n (%).

|  |  | Grade | | |
| --- | --- | --- | --- | --- |
|  |  | A | B | C |
| Stage | I | 0 (0) | 0 (0) | 0 (0) |
|  | II | 0 (0) | 2 (10) | 0 (0) |
|  | III | 0 (0) | 6 (30) | 7 (35) |
|  | IV | 0 (0) | 3 (15) | 2 (10) |

**Supplementary Table 6.** Analytical properties of quantification of bacterial target DNA in triplicate of 10-fold serially diluted standards using the multiplex quantitative real-time PCR (qPCR) and digital PCR (dPCR) assays.

|  | Estimated concentration (cp/reaction) | Multiplex qPCR assay - measured concentration | | | |  | Multiplex dPCR assay - measured concentration | | | | |
| --- | --- | --- | --- | --- | --- | --- | --- | --- | --- | --- | --- |
|  |  | Mean (cp/reaction) | SD | CV% | Accuracy (%) |  | Mean (cp/reaction) | SD | CV% | Accuracy (%) | λ |
| *Pg* | 1 × 10^6^ | 954423.93 | 33039.08 | 3.46 | 4.56 |  | Signal saturation | - | - | - | - |
|  | 1 × 10^5^ | 107579.26 | 10724.24 | 9.97 | 7.58 |  | 100281.33 | 2033.96 | 2.03 | 0.28 | 1.851 |
|  | 1 × 10^4^ | 10127.86 | 2201.36 | 21.74 | 1.28 |  | 9709.33 | 252.17 | 2.60 | 2.91 | 0.177 |
|  | 1 × 10^3^ | 979.78 | 71.75 | 7.32 | 2.02 |  | 876.27 | 29.94 | 3.42 | 12.37 | 0.016 |
|  | 1 × 10^2^ | 102.73 | 21.12 | 20.56 | 2.73 |  | 93.67 | 1.40 | 1.50 | 6.33 | 0.002 |
|  | 1 × 10^1^ | 10.32 | 3.54 | 34.33 | 3.24 |  | 13.20 | 5.94 | 45.02 | 32.00 | 0.0002 |
|  | 1 × 10^0^ | ND | - | - | - |  | ND | - | - | - | - |
|  | 1 × 10^-1^ | ND | - | - | - |  | ND | - | - | - | - |
| *Aa* | 1 × 10^6^ | 929359.22 | 62216.39 | 6.69 | 7.06 |  | Signal saturation | - | - | - | - |
|  | 1 × 10^5^ | 101891.14 | 7563.42 | 7.42 | 1.89 |  | 94260.00 | 2974.66 | 3.16 | 5.74 | 1.717 |
|  | 1 × 10^4^ | 9754.71 | 1413.55 | 14.49 | 2.45 |  | 9226.67 | 396.31 | 4.30 | 7.73 | 0.167 |
|  | 1 × 10^3^ | 1164.81 | 181.59 | 15.59 | 16.48 |  | 991.20 | 65.84 | 6.64 | 0.88 | 0.018 |
|  | 1 × 10^2^ | 112.60 | 12.98 | 11.53 | 12.60 |  | 85.72 | 6.72 | 7.84 | 14.28 | 0.002 |
|  | 1 × 10^1^ | 8.68 | 2.52 | 29.06 | 13.19 |  | 7.32 | 1.32 | 18.02 | 26.80 | 0.0001 |
|  | 1 × 10^0^ | ND | - | - | - |  | ND | - | - | - | - |
|  | 1 × 10^-1^ | ND | - | - | - |  | ND | - | - | - | - |
| *Fn* | 1 × 10^6^ | 1071382.17 | 104878.10 | 9.79 | 7.14 |  | Signal saturation | - | - | - | - |
|  | 1 × 10^5^ | 100215.99 | 6248.86 | 6.24 | 0.22 |  | 103722.67 | 4698.52 | 4.53 | 3.72 | 1.905 |
|  | 1 × 10^4^ | 9685.84 | 1110.68 | 11.47 | 3.14 |  | 9809.33 | 130.07 | 1.33 | 1.91 | 0.179 |
|  | 1 × 10^3^ | 961.87 | 54.11 | 5.63 | 3.81 |  | 972.00 | 68.35 | 7.03 | 2.80 | 0.018 |
|  | 1 × 10^2^ | 86.97 | 9.54 | 10.97 | 13.03 |  | 87.47 | 23.86 | 27.28 | 12.53 | 0.002 |
|  | 1 × 10^1^ | 11.89 | 2.74 | 23.02 | 18.88 |  | 10.83 | 2.00 | 18.48 | 8.27 | 0.0002 |
|  | 1 × 10^0^ | ND | - | - | - |  | ND | - | - | - | - |
|  | 1 × 10^-1^ | ND | - | - | - |  | ND | - | - | - | - |

*Pg*—*Porphyromonas gingivalis*; *Aa*—*Aggregatibacter actinomycetemcomitans*; Fn—*Fusobacterium nucleatum*; cp — copies; SD—standard deviation; CV%— coefficient of variation percentage; ND—not detected; λ—average number of targets per partition

**Supplementary Table 7.** Analytical properties of quantification of bacterial target DNA in triplicate of 10-fold serially diluted standards using the singlplex quantitative real-time PCR (qPCR) and digital PCR (dPCR) assays.

|  | Estimated concentration (cp/reaction) | Singleplex qPCR assays - measured concentration | | | |  | Singleplex dPCR assays - measured concentration | | | | |
| --- | --- | --- | --- | --- | --- | --- | --- | --- | --- | --- | --- |
|  |  | Mean (cp/reaction) | SD | CV% | Accuracy (%) |  | Mean (cp/reaction) | SD | CV% | Accuracy (%) | λ |
| *Pg* | 1 × 10^6^ | 1066395.62 | 92949.81 | 8.72 | 6.64 |  | Signal saturation | - | - | - | - |
|  | 1 × 10^5^ | 110115.70 | 18106.65 | 16.44 | 10.12 |  | 102420.00 | 4360.31 | 4.26 | 2.42 | 1.889 |
|  | 1 × 10^4^ | 9641.07 | 1889.13 | 19.59 | 3.59 |  | 9606.67 | 555.20 | 5.78 | 3.93 | 0.175 |
|  | 1 × 10^3^ | 865.41 | 65.11 | 7.52 | 13.46 |  | 899.47 | 91.89 | 10.22 | 10.05 | 0.016 |
|  | 1 × 10^2^ | 82.26 | 21.04 | 25.57 | 17.74 |  | 89.84 | 2.60 | 2.89 | 10.16 | 0.002 |
|  | 1 × 10^1^ | 13.34 | 3.75 | 28.09 | 33.43 |  | 12.33 | 3.22 | 26.09 | 23.33 | 0.0002 |
|  | 1 × 10^0^ | ND | - | - | - |  | ND | - | - | - | - |
|  | 1 × 10^-1^ | ND | - | - | - |  | ND | - | - | - | - |
| *Aa* | 1 × 10^6^ | 1166499.64 | 61022.20 | 5.23 | 16.65 |  | Signal saturation | - | - | - | - |
|  | 1 × 10^5^ | 102520.92 | 11250.08 | 10.97 | 2.52 |  | 104173.33 | 2423.63 | 2.33 | 4.17 | 1.914 |
|  | 1 × 10^4^ | 9279.00 | 1061.25 | 11.44 | 7.21 |  | 9570.67 | 170.10 | 1.78 | 4.29 | 0.174 |
|  | 1 × 10^3^ | 994.16 | 102.03 | 10.26 | 0.58 |  | 925.87 | 82.72 | 8.93 | 7.41 | 0.017 |
|  | 1 × 10^2^ | 81.07 | 7.94 | 9.80 | 18.93 |  | 101.47 | 10.10 | 9.96 | 1.47 | 0.002 |
|  | 1 × 10^1^ | 8.13 | 2.73 | 33.60 | 18.68 |  | 12.25 | 2.31 | 18.86 | 22.53 | 0.0002 |
|  | 1 × 10^0^ | 1.59 | 0.82 | 51.92 | 58.66 |  | ND | - | - | - | - |
|  | 1 × 10^-1^ | ND | - | - | - |  | ND | - | - | - | - |
| *Fn* | 1 × 10^6^ | 1064165.12 | 51514.08 | 4.84 | 6.42 |  | Signal saturation | - | - | - | - |
|  | 1 × 10^5^ | 106868.88 | 9272.87 | 8.68 | 6.87 |  | 103380.00 | 2255.08 | 2.18 | 3.38 | 1.908 |
|  | 1 × 10^4^ | 9085.18 | 1078.49 | 11.87 | 9.15 |  | 10082.67 | 348.81 | 3.46 | 0.83 | 0.184 |
|  | 1 × 10^3^ | 960.42 | 120.60 | 12.56 | 3.96 |  | 993.07 | 55.24 | 5.56 | 0.69 | 0.018 |
|  | 1 × 10^2^ | 84.79 | 9.29 | 10.96 | 15.21 |  | 75.23 | 16.50 | 21.93 | 24.77 | 0.001 |
|  | 1 × 10^1^ | 13.01 | 6.22 | 47.79 | 30.06 |  | 8.03 | 1.37 | 17.12 | 19.73 | 0.0001 |
|  | 1 × 10^0^ | ND | - | - | - |  | ND | - | - | - | - |
|  | 1 × 10^-1^ | ND | - | - | - |  | ND | - | - | - | - |

*Pg*—*Porphyromonas gingivalis*; *Aa*—*Aggregatibacter actinomycetemcomitans*; Fn—*Fusobacterium nucleatum*; cp — copies; SD—standard deviation; CV%— coefficient of variation percentage; ND—not detected; λ—average number of targets per partition

**Supplementary Table 8.** Data from the testing of clinical samples of periodontal patients (1-20) and healthy controls (21-40) and their dilutions using the multiplex digital PCR assay (determined DNA concentration, dilution factor, confidence interval of 95%, number of partitions for each sample tested, and average number of targets per partition (λ)).

| **Sample Number** | **Ct Value (qPCR)** | **Dilution Factor** | **Bacteria** | **DNA Concentration (cp/µL)** | **CI (95%)** | **Valid Partitions** | **Positive Partitions** | **Negative Partitions** | **λ** | **Calculated DNA Concentration in Original Sample (cp/µL)** |
| --- | --- | --- | --- | --- | --- | --- | --- | --- | --- | --- |
| f | negative | 10^0  (original sample) | *A. actinomycetemcomitans* | 2.504 | 56.60% | 25419 | 12 | 25407 | 0.0005 | 2.504 |
|  | 21.485 |  | *P. gingivalis* | signal saturation | - | 25419 | 25418 | 1 | - | 50928.00 |
|  | 20.302 |  | *F. nucleatum* | signal saturation | - | 25419 | 25419 | 0 | - | 87428.00 |
|  |  | 10^1 | *A. actinomycetemcomitans* | negative | - | 25362 | 0 | 25362 | - |  |
|  |  |  | *P. gingivalis* | 5165.60 | 1.60% | 25362 | 15166 | 10196 | 0.9113 |  |
|  |  |  | *F. nucleatum* | 8865.00 | 1.50% | 25362 | 20054 | 5308 | 1.5640 |  |
|  |  | 10^2 | *A. actinomycetemcomitans* | negative | - | 25436 | 0 | 25436 | - |  |
|  |  |  | *P. gingivalis* | 502.00 | 4.10% | 25436 | 2266 | 23158 | 0.0933 |  |
|  |  |  | *F. nucleatum* | 862.00 | 3.20% | 25436 | 3762 | 21634 | 0.1601 |  |
| 2 | negative | 10^0  (original sample) | *A. actinomycetemcomitans* | 1.704 | 69.30% | 25438 | 8 | 25430 | 0.0003 | 1.704 |
|  | 18.689 |  | *P. gingivalis* | signal saturation | - | 25438 | 25436 | 2 | - | 300160.00 |
|  | 18.336 |  | *F. nucleatum* | signal saturation | - | 25438 | 25435 | 3 | - | 363960.00 |
|  |  | 10^2 | *A. actinomycetemcomitans* | negative | - | 25415 | 0 | 25415 | - |  |
|  |  |  | *P. gingivalis* | 3145.60 | 1.90% | 25415 | 10885 | 14530 | 0.5591 |  |
|  |  |  | *F. nucleatum* | 3713.20 | 1.80% | 25415 | 12280 | 13135 | 0.6601 |  |
|  |  | 10^3 | *A. actinomycetemcomitans* | negative | - | 25456 | 0 | 25456 | - |  |
|  |  |  | *P. gingivalis* | 285.76 | 5.40% | 25450 | 1305 | 24145 | 0.0526 |  |
|  |  |  | *F. nucleatum* | 356.60 | 4.90% | 25450 | 1618 | 23832 | 0.0657 |  |
| 3 | negative | 10^0  (original sample) | *A. actinomycetemcomitans* | 2.648 | 56.60% | 25436 | 12 | 25424 | 0.0005 | 2.648 |
|  | 31.41 |  | *P. gingivalis* | 89.00 | 9.80% | 25430 | 400 | 25030 | 0.0159 | 89.00 |
|  | 20.417 |  | *F. nucleatum* | signal saturation | - | 25436 | 25433 | 3 | - | 84660.00 |
|  |  | 10^2 | *A. actinomycetemcomitans* | negative | - | 25392 | 0 | 25392 | - |  |
|  |  |  | *P. gingivalis* | 0.652 | 106.40% | 25392 | 3 | 25389 | 0.0001 |  |
|  |  |  | *F. nucleatum* | 882.40 | 3.20% | 25305 | 3749 | 21556 | 0.1603 |  |
|  |  | 10^3 | *A. actinomycetemcomitans* | negative | - | 25411 | 0 | 25411 | - |  |
|  |  |  | *P. gingivalis* | negative | - | 25411 | 0 | 25411 | - |  |
|  |  |  | *F. nucleatum* | 81.08 | 10.10% | 25411 | 375 | 25036 | 0.0149 |  |
| 4 | 40.06 | 10^0  (original sample) | *A. actinomycetemcomitans* | 1.340 | 80.00% | 25428 | 6 | 25422 | 0.0002 | 1.340 |
|  | 32.354 |  | *P. gingivalis* | 51.44 | 13.00% | 25428 | 229 | 25199 | 0.0090 | 51.44 |
|  | 18.704 |  | *F. nucleatum* | signal saturation | - | 25428 | 25428 | 0 | - | 258960.00 |
|  |  | 10^2 | *A. actinomycetemcomitans* | negative | - | 25360 | 0 | 25360 | - |  |
|  |  |  | *P. gingivalis* | 0.872 | 98.00% | 25360 | 4 | 25356 | 0.0002 |  |
|  |  |  | *F. nucleatum* | 2539.60 | 2.10% | 25112 | 9258 | 15854 | 0.4599 |  |
|  |  | 10^3 | *A. actinomycetemcomitans* | negative | - | 25410 | 0 | 25410 | - |  |
|  |  |  | *P. gingivalis* | negative | - | 25410 | 0 | 25410 | - |  |
|  |  |  | *F. nucleatum* | 263.96 | 5.20% | 25410 | 1200 | 24210 | 0.0484 |  |
| 5 | 42.753 | 10^0  (original sample) | *A. actinomycetemcomitans* | 1.736 | 69.30% | 25415 | 8 | 25407 | 0.0003 | 1.736 |
|  | 32.971 |  | *P. gingivalis* | 41.36 | 14.20% | 25413 | 190 | 25223 | 0.0075 | 41.36 |
|  | 20.678 |  | *F. nucleatum* | signal saturation | - | 25415 | 25415 | 0 | - | 76880.00 |
|  |  | 10^2 | *A. actinomycetemcomitans* | negative | - | 25417 | 0 | 25417 | - |  |
|  |  |  | *P. gingivalis* | negative | - | 25417 | 1 | 25416 | - |  |
|  |  |  | *F. nucleatum* | 756.40 | 3.40% | 25389 | 3284 | 22105 | 0.1385 |  |
|  |  | 10^3 | *A. actinomycetemcomitans* | negative | - | 25372 | 1 | 25371 | - |  |
|  |  |  | *P. gingivalis* | negative | - | 25372 | 1 | 25371 | - |  |
|  |  |  | *F. nucleatum* | 78.12 | 10.50% | 25372 | 349 | 25023 | 0.0139 |  |
| 6 | 32.373 | 10^0  (original sample) | *A. actinomycetemcomitans* | 263.52 | 5.70% | 25274 | 1171 | 24097 | 0.0474 | 263.52 |
|  | 20.73 |  | *P. gingivalis* | signal saturation | - | 25321 | 25321 | 0 | - | 103610.00 |
|  | 20.592 |  | *F. nucleatum* | signal saturation | - | 25321 | 25321 | 0 | - | 83152.00 |
|  |  | 10^1 | *A. actinomycetemcomitans* | 23.45 | 18.90% | 25436 | 108 | 25328 | 0.0043 |  |
|  |  |  | *P. gingivalis* | 9962.00 | 1.50% | 25438 | 21264 | 4174 | 1.8074 |  |
|  |  |  | *F. nucleatum* | 8098.40 | 1.50% | 25438 | 19585 | 5853 | 1.4693 |  |
|  |  | 10^2 | *A. actinomycetemcomitans* | 1.724 | 69.30% | 25420 | 8 | 25412 | 0.0003 |  |
|  |  |  | *P. gingivalis* | 1076.00 | 2.90% | 25330 | 4518 | 20812 | 0.1965 |  |
|  |  |  | *F. nucleatum* | 853.20 | 3.20% | 25398 | 3664 | 21734 | 0.1558 |  |
| 7 | negative | 10^0  (original sample) | *A. actinomycetemcomitans* | negative | - | 25456 | 1 | 25455 | - | - |
|  | 27.932 |  | *P. gingivalis* | 1143.20 | 2.80% | 25309 | 4906 | 20403 | 0.2155 | 1143.20 |
|  | 27.055 |  | *F. nucleatum* | 1609.20 | 2.40% | 25321 | 6624 | 18697 | 0.3033 | 1609.20 |
| 8 | negative | 10^0  (original sample) | *A. actinomycetemcomitans* | 1.056 | 87.70% | 25459 | 5 | 25454 | 0.0002 | 1.056 |
|  | 21.119 |  | *P. gingivalis* | signal saturation | - | 25459 | 25458 | 1 | - | 83194.00 |
|  | 21.154 |  | *F. nucleatum* | signal saturation | - | 25459 | 25452 | 7 | - | 56300.00 |
|  |  | 10^1 | *A. actinomycetemcomitans* | negative | - | 25397 | 0 | 25397 | - |  |
|  |  |  | *P. gingivalis* | 8178.80 | 1.50% | 25397 | 19900 | 5497 | 1.5304 |  |
|  |  |  | *F. nucleatum* | 5708.00 | 1.60% | 25397 | 16669 | 8728 | 1.0681 |  |
|  |  | 10^2 | *A. actinomycetemcomitans* | negative | - | 25310 | 1 | 25309 | - |  |
|  |  |  | *P. gingivalis* | 846.00 | 3.20% | 25278 | 3656 | 21622 | 0.1562 |  |
|  |  |  | *F. nucleatum* | 555.20 | 3.90% | 25301 | 2465 | 22836 | 0.1025 |  |
| 9 | 22.736 | 10^0  (original sample) | *A. actinomycetemcomitans* | 5447.20 | 1.60% | 25415 | 15980 | 9435 | 0.9909 | 5447.20 |
|  | 19.2 |  | *P. gingivalis* | signal saturation | - | 25415 | 25412 | 3 | - | 231730.00 |
|  | 18.734 |  | *F. nucleatum* | signal saturation | - | 25415 | 25412 | 3 | - | 253526.00 |
|  |  | 10^1 | *A. actinomycetemcomitans* | 527.60 | 4.10% | 25449 | 2343 | 23106 | 0.0966 |  |
|  |  |  | *P. gingivalis* | 22974.00 | 2.40% | 25458 | 25079 | 379 | 4.2072 |  |
|  |  |  | *F. nucleatum* | 24765.20 | 2.60% | 25458 | 25185 | 273 | 4.5353 |  |
|  |  | 10^2 | *A. actinomycetemcomitans* | 57.12 | 11.90% | 25416 | 272 | 25144 | 0.0108 |  |
|  |  |  | *P. gingivalis* | 2337.20 | 2.10% | 25147 | 8953 | 16194 | 0.4401 |  |
|  |  |  | *F. nucleatum* | 2594.00 | 2.00% | 25188 | 9734 | 15454 | 0.4885 |  |
| 10 | negative | 10^0  (original sample) | *A. actinomycetemcomitans* | 1.932 | 65.30% | 25314 | 9 | 25305 | 0.0004 | 1.932 |
|  | 20.352 |  | *P. gingivalis* | signal saturation | - | 25314 | 25306 | 8 | - | 142080.00 |
|  | 19.377 |  | *F. nucleatum* | signal saturation | - | 25314 | 25305 | 9 | - | 186920.00 |
|  |  | 10^2 | *A. actinomycetemcomitans* | negative | - | 25447 | 0 | 25447 | - |  |
|  |  |  | *P. gingivalis* | 1445.60 | 2.60% | 25280 | 5877 | 19403 | 0.2646 |  |
|  |  |  | *F. nucleatum* | 1904.00 | 2.30% | 25264 | 7434 | 17830 | 0.3485 |  |
|  |  | 10^3 | *A. actinomycetemcomitans* | negative | - | 25442 | 0 | 25442 | - |  |
|  |  |  | *P. gingivalis* | 139.60 | 7.80% | 25442 | 625 | 24817 | 0.0249 |  |
|  |  |  | *F. nucleatum* | 183.44 | 6.90% | 25440 | 818 | 24622 | 0.0327 |  |
| 11 | negative | 10^0  (original sample) | *A. actinomycetemcomitans* | 1.512 | 74.10% | 25452 | 7 | 25445 | 0.0003 | 1.512 |
|  | 21.406 |  | *P. gingivalis* | signal saturation | - | 25452 | 25452 | 0 | - | 57480.00 |
|  | 20.659 |  | *F. nucleatum* | signal saturation | - | 25452 | 25452 | 0 | - | 72380.00 |
|  |  | 10^2 | *A. actinomycetemcomitans* | negative | - | 25286 | 0 | 25286 | - |  |
|  |  |  | *P. gingivalis* | 628.80 | 3.70% | 25274 | 2752 | 22522 | 0.1153 |  |
|  |  |  | *F. nucleatum* | 718.80 | 3.50% | 25274 | 3122 | 22152 | 0.1318 |  |
|  |  | 10^3 | *A. actinomycetemcomitans* | negative | - | 25436 | 0 | 25436 | - |  |
|  |  |  | *P. gingivalis* | 52.08 | 12.90% | 25436 | 232 | 25204 | 0.0092 |  |
|  |  |  | *F. nucleatum* | 72.88 | 10.90% | 25436 | 324 | 25112 | 0.0128 |  |
| 12 | negative | 10^0  (original sample) | *A. actinomycetemcomitans* | negative | - | 25421 | 0 | 25421 | - | - |
|  | 31.828 |  | *P. gingivalis* | 86.68 | 9.80% | 25421 | 396 | 25025 | 0.0157 | 86.68 |
|  | 19.203 |  | *F. nucleatum* | signal saturation | - | 25421 | 25420 | 1 | - | 208580.00 |
|  |  | 10^2 | *A. actinomycetemcomitans* | negative | - | 25395 | 0 | 25395 | - |  |
|  |  |  | *P. gingivalis* | negative | - | 25395 | 2 | 25393 | - |  |
|  |  |  | *F. nucleatum* | 2145.60 | 2.20% | 25099 | 8160 | 16939 | 0.3932 |  |
|  |  | 10^3 | *A. actinomycetemcomitans* | negative | - | 25413 | 0 | 25413 | - |  |
|  |  |  | *P. gingivalis* | negative | - | 25413 | 0 | 25413 | - |  |
|  |  |  | *F. nucleatum* | 202.60 | 6.60% | 25407 | 892 | 24515 | 0.0357 |  |
| 13 | negative | 10^0  (original sample) | *A. actinomycetemcomitans* | 1.064 | 87.70% | 24962 | 5 | 24957 | 0.0002 | 1.064 |
|  | 32.849 |  | *P. gingivalis* | 51.24 | 12.70% | 24962 | 240 | 24722 | 0.0097 | 51.24 |
|  | 27.332 |  | *F. nucleatum* | 941.60 | 3.10% | 24917 | 4052 | 20865 | 0.1775 | 941.60 |
| 14 | negative | 10^0  (original sample) | *A. actinomycetemcomitans* | negative | - | 25426 | 1 | 25425 | - | - |
|  | 26.628 |  | *P. gingivalis* | 2064.00 | 2.20% | 25239 | 7883 | 17356 | 0.3745 | 2118.00 |
|  | 21.206 |  | *F. nucleatum* | signal saturation | - | 25426 | 25423 | 3 | - | 52420.00 |
|  |  | 10^2 | *A. actinomycetemcomitans* | negative | - | 25444 | 0 | 25444 | - |  |
|  |  |  | *P. gingivalis* | 21.72 | 19.50% | 25444 | 101 | 25343 | 0.0040 |  |
|  |  |  | *F. nucleatum* | 518.40 | 4.10% | 25444 | 2305 | 23139 | 0.0950 |  |
|  |  | 10^3 | *A. actinomycetemcomitans* | negative | - | 25345 | 0 | 25345 | - |  |
|  |  |  | *P. gingivalis* | 1.560 | 74.10% | 25345 | 7 | 25338 | 0.0003 |  |
|  |  |  | *F. nucleatum* | 53.00 | 12.70% | 25345 | 237 | 25108 | 0.0094 |  |
| 15 | negative | 10^0  (original sample) | *A. actinomycetemcomitans* | 0.872 | 98.00% | 25392 | 4 | 25388 | 0.0002 | 0.872 |
|  | 32.764 |  | *P. gingivalis* | 41.30 | 14.60% | 25392 | 181 | 25211 | 0.0072 | 41.30 |
|  | 22.061 |  | *F. nucleatum* | 26877.60 | 2.90% | 25392 | 25196 | 196 | 4.8641 | 26877.60 |
| 16 | negative | 10^0  (original sample) | *A. actinomycetemcomitans* | 1.300 | 80.00% | 25384 | 6 | 25378 | 0.0002 | 1.300 |
|  | 21.557 |  | *P. gingivalis* | signal saturation | - | 25384 | 25383 | 1 | - | 61780.00 |
|  | 20.159 |  | *F. nucleatum* | signal saturation | - | 25384 | 25383 | 1 | - | 102960.00 |
|  |  | 10^2 | *A. actinomycetemcomitans* | negative | - | 25224 | 0 | 25224 | - |  |
|  |  |  | *P. gingivalis* | 608.80 | 3.80% | 25224 | 2732 | 22492 | 0.1146 |  |
|  |  |  | *F. nucleatum* | 1072.00 | 2.90% | 25139 | 4595 | 20544 | 0.2019 |  |
|  |  | 10^3 | *A. actinomycetemcomitans* | negative | - | 25418 | 0 | 25418 | - |  |
|  |  |  | *P. gingivalis* | 62.68 | 11.50% | 25418 | 293 | 25125 | 0.0116 |  |
|  |  |  | *F. nucleatum* | 98.72 | 9.10% | 25418 | 460 | 24958 | 0.0183 |  |
| 17 | 24.543 | 10^0  (original sample) | *A. actinomycetemcomitans* | 1472.00 | 2.50% | 25167 | 6028 | 19139 | 0.2738 | 1472.00 |
|  | 20.685 |  | *P. gingivalis* | signal saturation | - | 25464 | 25459 | 5 | - | 99430.00 |
|  | 20.215 |  | *F. nucleatum* | signal saturation | - | 25464 | 25458 | 6 | - | 93122.00 |
|  |  | 10^1 | *A. actinomycetemcomitans* | 155.28 | 7.50% | 25412 | 690 | 24722 | 0.0275 |  |
|  |  |  | *P. gingivalis* | 10166.00 | 1.50% | 25414 | 21223 | 4191 | 1.8024 |  |
|  |  |  | *F. nucleatum* | 9292.40 | 1.50% | 25414 | 20521 | 4893 | 1.6475 |  |
|  |  | 10^2 | *A. actinomycetemcomitans* | 13.56 | 24.50% | 25244 | 64 | 25180 | 0.0025 |  |
|  |  |  | *P. gingivalis* | 972.00 | 3.00% | 25178 | 4187 | 20991 | 0.1819 |  |
|  |  |  | *F. nucleatum* | 933.20 | 3.10% | 25231 | 4043 | 21188 | 0.1746 |  |
| 18 | negative | 10^0  (original sample) | *A. actinomycetemcomitans* | negative | - | 25256 | 0 | 25256 | - | - |
|  | 20.385 |  | *P. gingivalis* | signal saturation | - | 25256 | 25251 | 5 | - | 126360.00 |
|  | 20.118 |  | *F. nucleatum* | signal saturation | - | 25256 | 25250 | 6 | - | 103600.00 |
|  |  | 10^2 | *A. actinomycetemcomitans* | negative | - | 25339 | 0 | 25339 | - |  |
|  |  |  | *P. gingivalis* | 1244.80 | 2.70% | 25165 | 5157 | 20008 | 0.2293 |  |
|  |  |  | *F. nucleatum* | 999.20 | 3.00% | 25280 | 4250 | 21030 | 0.1841 |  |
|  |  | 10^3 | *A. actinomycetemcomitans* | negative | - | 25355 | 1 | 25354 | - |  |
|  |  |  | *P. gingivalis* | 128.24 | 8.10% | 25347 | 588 | 24759 | 0.0235 |  |
|  |  |  | *F. nucleatum* | 107.28 | 8.80% | 25355 | 493 | 24862 | 0.0196 |  |
| 19 | negative | 10^0  (original sample) | *A. actinomycetemcomitans* | 2.652 | 56.60% | 25393 | 12 | 25381 | 0.0005 | 2.652 |
|  | 27.342 |  | *P. gingivalis* | 1265.20 | 2.70% | 25279 | 5102 | 20177 | 0.2254 | 1265.20 |
|  | 19.851 |  | *F. nucleatum* | signal saturation | - | 25400 | 25398 | 2 | - | 140460.00 |
|  |  | 10^2 | *A. actinomycetemcomitans* | negative | - | 25385 | 0 | 25385 | - |  |
|  |  |  | *P. gingivalis* | 13.024 | 25.30% | 25385 | 60 | 25325 | 0.0024 |  |
|  |  |  | *F. nucleatum* | 1400.40 | 2.60% | 25141 | 5648 | 19493 | 0.2544 |  |
|  |  | 10^3 | *A. actinomycetemcomitans* | negative | - | 25381 | 0 | 25381 | - |  |
|  |  |  | *P. gingivalis* | 0.644 | 106.40% | 25381 | 3 | 25378 | 0.0001 |  |
|  |  |  | *F. nucleatum* | 140.88 | 7.70% | 25369 | 647 | 24722 | 0.0258 |  |
| 20 | negative | 10^0  (original sample) | *A. actinomycetemcomitans* | 1.116 | 87.70% | 25442 | 5 | 25437 | 0.0002 | 1.116 |
|  | 18.321 |  | *P. gingivalis* | signal saturation | - | 25442 | 25439 | 3 | - | 554380.00 |
|  | 19.292 |  | *F. nucleatum* | signal saturation | - | 25442 | 25436 | 6 | - | 180720.00 |
|  |  | 10^2 | *A. actinomycetemcomitans* | negative | - | 25428 | 0 | 25428 | - |  |
|  |  |  | *P. gingivalis* | 5495.60 | 1.60% | 25428 | 16030 | 9398 | 0.9954 |  |
|  |  |  | *F. nucleatum* | 1816.40 | 2.30% | 25282 | 7087 | 18195 | 0.3289 |  |
|  |  | 10^3 | *A. actinomycetemcomitans* | negative | - | 25336 | 0 | 25336 | - |  |
|  |  |  | *P. gingivalis* | 559.20 | 3.90% | 25317 | 2466 | 22851 | 0.1025 |  |
|  |  |  | *F. nucleatum* | 179.80 | 6.80% | 25330 | 821 | 24509 | 0.0329 |  |
| 21 | negative | 10^0  (original sample) | *A. actinomycetemcomitans* | negative | - | 25378 | 2 | 25376 | - | - |
|  | 36.331 |  | *P. gingivalis* | negative | - | 25378 | 2 | 25376 | - | - |
|  | 25.771 |  | *F. nucleatum* | 3291.60 | 1.90% | 25378 | 11411 | 13967 | 0.5972 | 3291.60 |
| 22 | 40.151 | 10^0  (original sample) | *A. actinomycetemcomitans* | negative | - | 25365 | 2 | 25363 | - | - |
|  | 33.539 |  | *P. gingivalis* | 1.336 | 80.00% | 25365 | 6 | 25359 | 0.0002 | 1.336 |
|  | 26.509 |  | *F. nucleatum* | 1641.20 | 2.50% | 25238 | 6372 | 18866 | 0.2910 | 1641.20 |
| 23 | negative | 10^0  (original sample) | *A. actinomycetemcomitans* | 0.868 | 98.00% | 25250 | 4 | 25246 | 0.0002 | 0.868 |
|  | 36.503 |  | *P. gingivalis* | 1.084 | 87.70% | 25250 | 5 | 25245 | 0.0002 | 1.084 |
|  | 25.28 |  | *F. nucleatum* | 4576.40 | 1.70% | 25250 | 14302 | 10948 | 0.8357 | 4576.40 |
| 24 | 27.755 | 10^0  (original sample) | *A. actinomycetemcomitans* | 114.96 | 8.60% | 25371 | 525 | 24846 | 0.0209 | 114.96 |
|  | 31.92 |  | *P. gingivalis* | negative | - | 25391 | 2 | 25389 | - | - |
|  | 31.955 |  | *F. nucleatum* | 56.60 | 12.20% | 25386 | 260 | 25126 | 0.0103 | 56.60 |
| 25 | negative | 10^0  (original sample) | *A. actinomycetemcomitans* | negative | - | 25079 | 2 | 25077 | - | - |
|  | 37.202 |  | *P. gingivalis* | negative | - | 25079 | 1 | 25078 | - | - |
|  | 30.948 |  | *F. nucleatum* | 94.20 | 9.30% | 25079 | 441 | 24638 | 0.0177 | 94.20 |
| 26 | negative | 10^0  (original sample) | *A. actinomycetemcomitans* | 1.460 | 74.10% | 25415 | 7 | 25408 | 0.0003 | 1.460 |
|  | 39.518 |  | *P. gingivalis* | 1.252 | 80.00% | 25415 | 6 | 25409 | 0.0002 | 1.252 |
|  | 21.538 |  | *F. nucleatum* | signal saturation | - | 25415 | 25414 | 1 | - | 56272.00 |
|  |  | 10^1 | *A. actinomycetemcomitans* | 0.632 | 106.40% | 25439 | 3 | 25436 | 0.0001 |  |
|  |  |  | *P. gingivalis* | negative | - | 25439 | 0 | 25439 | - |  |
|  |  |  | *F. nucleatum* | 5758.40 | 1.60% | 25439 | 16724 | 8715 | 1.0712 |  |
|  |  | 10^2 | *A. actinomycetemcomitans* | negative | - | 25388 | 1 | 25387 | - |  |
|  |  |  | *P. gingivalis* | negative | - | 25388 | 0 | 25388 | - |  |
|  |  |  | *F. nucleatum* | 549.60 | 3.90% | 25385 | 2481 | 22904 | 0.1028 |  |
| 27 | negative | 10^0  (original sample) | *A. actinomycetemcomitans* | negative | - | 25425 | 0 | 25425 | - | - |
|  | 37.24 |  | *P. gingivalis* | negative | - | 25425 | 2 | 25423 | - | - |
|  | 28.711 |  | *F. nucleatum* | 500.80 | 4.10% | 25419 | 2249 | 23170 | 0.0926 | 500.80 |
| 28 | negative | 10^0  (original sample) | *A. actinomycetemcomitans* | negative | - | 25424 | 0 | 25424 | - | - |
|  | negative |  | *P. gingivalis* | negative | - | 25424 | 2 | 25422 | - | - |
|  | 23.924 |  | *F. nucleatum* | 11876.00 | 1.60% | 25424 | 22345 | 3079 | 2.1111 | 11876.00 |
| 29 | 37.5 | 10^0  (original sample) | *A. actinomycetemcomitans* | negative | - | 25413 | 2 | 25411 | - | - |
|  | 34.492 |  | *P. gingivalis* | 0.884 | 98.00% | 25413 | 4 | 25413 | 0.0002 | 0.884 |
|  | 24.699 |  | *F. nucleatum* | 5663.60 | 1.60% | 25413 | 16148 | 9265 | 1.0090 | 5663.60 |
| 30 | 21.21 | 10^0  (original sample) | *A. actinomycetemcomitans* | 10578.00 | 1.50% | 25433 | 21778 | 3655 | 1.9400 | 10578.00 |
|  | 21.211 |  | *P. gingivalis* | 7584.00 | 1.50% | 25433 | 19104 | 6329 | 1.3909 | 7584.00 |
|  | 21.773 |  | *F. nucleatum* | signal saturation | - | 25433 | 25423 | 10 | - | 42788.00 |
|  |  | 10^1 | *A. actinomycetemcomitans* | 1042.40 | 3.00% | 25424 | 4260 | 21164 | 0.1834 |  |
|  |  |  | *P. gingivalis* | 758.80 | 3.50% | 25440 | 3178 | 22262 | 0.1334 |  |
|  |  |  | *F. nucleatum* | 4357.60 | 1.70% | 25458 | 13629 | 11829 | 0.7665 |  |
|  |  | 10^2 | *A. actinomycetemcomitans* | 95.00 | 9.40% | 25444 | 434 | 25010 | 0.0172 |  |
|  |  |  | *P. gingivalis* | 75.56 | 10.50% | 25452 | 346 | 25106 | 0.0137 |  |
|  |  |  | *F. nucleatum* | 420.00 | 4.50% | 25446 | 1864 | 23582 | 0.0761 |  |
| 31 | 33.667 | 10^0  (original sample) | *A. actinomycetemcomitans* | 5.128 | 40.90% | 25436 | 23 | 25413 | 0.0009 | 5.128 |
|  | 35.578 |  | *P. gingivalis* | 1.560 | 74.10% | 25436 | 7 | 25429 | 0.0003 | 1.560 |
|  | 23.23 |  | *F. nucleatum* | 15478.00 | 1.70% | 25436 | 23778 | 1658 | 2.7306 | 15478.00 |
| 32 | 28.96 | 10^0  (original sample) | *A. actinomycetemcomitans* | 39.85 | 14.60% | 25427 | 179 | 25248 | 0.0071 | 39.85 |
|  | 31.567 |  | *P. gingivalis* | 8.436 | 31.80% | 25427 | 38 | 25389 | 0.0015 | 8.436 |
|  | 30.736 |  | *F. nucleatum* | 112.92 | 8.70% | 25427 | 504 | 24923 | 0.0200 | 112.92 |
| 33 | negative | 10^0  (original sample) | *A. actinomycetemcomitans* | negative | - | 25397 | 1 | 25396 | - | - |
|  | negative |  | *P. gingivalis* | negative | - | 25397 | 2 | 25395 | - | - |
|  | 24.604 |  | *F. nucleatum* | 7641.20 | 1.50% | 25397 | 19026 | 6371 | 1.3829 | 7641.20 |
| 34 | negative | 10^0  (original sample) | *A. actinomycetemcomitans* | negative | - | 25232 | 2 | 25230 | - | - |
|  | 28.187 |  | *P. gingivalis* | 161.04 | 7.30% | 25232 | 722 | 24510 | 0.0290 | 161.04 |
|  | 27.976 |  | *F. nucleatum* | 688.80 | 3.60% | 25212 | 2944 | 22268 | 0.1242 | 688.80 |
| 35 | 35.255 | 10^0  (original sample) | *A. actinomycetemcomitans* | 1.044 | 87.70% | 25437 | 5 | 25432 | 0.0002 | 1.044 |
|  | 34.953 |  | *P. gingivalis* | negative | - | 25437 | 1 | 25436 | - | - |
|  | 24.25 |  | *F. nucleatum* | 6572.00 | 1.50% | 25437 | 18908 | 6529 | 1.3600 | 6572.00 |
| 36 | 22.147 | 10^0  (original sample) | *A. actinomycetemcomitans* | 5088.00 | 1.60% | 25366 | 15314 | 10052 | 0.9256 | 5088.00 |
|  | 30.266 |  | *P. gingivalis* | negative | - | 25366 | 2 | 24364 | - | - |
|  | 23.755 |  | *F. nucleatum* | 11053.20 | 1.60% | 25366 | 21970 | 3396 | 2.0108 | 11053.20 |
| 37 | 32.448 | 10^0  (original sample) | *A. actinomycetemcomitans* | 13.30 | 24.70% | 25353 | 63 | 25290 | 0.0025 | 13.296 |
|  | 29.534 |  | *P. gingivalis* | 43.80 | 13.60% | 25353 | 207 | 25146 | 0.0082 | 43.80 |
|  | 25.031 |  | *F. nucleatum* | 4920.80 | 1.60% | 25353 | 15257 | 10096 | 0.9208 | 4920.80 |
| 38 | negative | 10^0  (original sample) | *A. actinomycetemcomitans* | 3.976 | 46.20% | 25467 | 18 | 25449 | 0.0007 | 3.976 |
|  | 25.953 |  | *P. gingivalis* | 527.20 | 4.10% | 25467 | 2278 | 23189 | 0.0937 | 527.20 |
|  | 23.124 |  | *F. nucleatum* | 16710.40 | 1.80% | 25136 | 23847 | 1289 | 2.9704 | 16710.40 |
| 39 | 29.972 | 10^0  (original sample) | *A. actinomycetemcomitans* | 21.65 | 19.80% | 25459 | 98 | 25361 | 0.0039 | 21.65 |
|  | 33.477 |  | *P. gingivalis* | 4.632 | 42.80% | 25459 | 21 | 25438 | 0.0008 | 4.632 |
|  | 21.587 |  | *F. nucleatum* | signal saturation | - | 25459 | 25453 | 6 | - | 62628.00 |
|  |  | 10^1 | *A. actinomycetemcomitans* | 2.824 | 54.40% | 25344 | 13 | 25331 | 0.0005 |  |
|  |  |  | *P. gingivalis* | 0.868 | 98.00% | 25344 | 4 | 25340 | 0.0002 |  |
|  |  |  | *F. nucleatum* | 6129.60 | 1.60% | 25344 | 17022 | 8322 | 1.1136 |  |
|  |  | 10^2 | *A. actinomycetemcomitans* | negative | - | 25404 | 1 | 25403 | - |  |
|  |  |  | *P. gingivalis* | negative | - | 25404 | 0 | 25404 | - |  |
|  |  |  | *F. nucleatum* | 639.60 | 3.70% | 25372 | 2808 | 22564 | 0.1173 |  |
| 40 | negative | 10^0  (original sample) | *A. actinomycetemcomitans* | negative | - | 25372 | 1 | 25371 | - | - |
|  | 35.426 |  | *P. gingivalis* | 0.872 | 98.00% | 25372 | 4 | 25368 | 0.0002 | 0.872 |
|  | 25.884 |  | *F. nucleatum* | 3094.80 | 1.90% | 25372 | 10887 | 14485 | 0.5605 | 3094.80 |

**Supplementary table 9.** Diagnostic parameters for *Porphyromonas gingivalis*, *Aggregatibacter actinomycetemcomitans*, and *Fusobacterium nucleatum* using the multiplex quantitative real-time PCR (qPCR) assay or the multiplex digital PCR (dPCR) assay.

|  |  | qPCR | |  | dPCR | |
| --- | --- | --- | --- | --- | --- | --- |
|  |  | Value | 95% CI |  | Value | 95% CI |
|  |  |  |  |  |  |  |
| *Pg* | Sensitivity | 1.00 | 0.84 − 1.00 |  | 1.00 | 0.84 − 1.00 |
|  | Specificity | 0.75 | 0.53 − 0.89 |  | 0.40 | 0.22 − 0.61 |
|  | Positive Predictive Value | 0.80 | 0.61 − 0.91 |  | 0.63 | 0.45 − 0.77 |
|  | Negative Predictive Value | 1.00 | 0.80 − 1.00 |  | 1.00 | 0.68 − 1.00 |
|  |  |  |  |  |  |  |
|  |  |  |  |  |  |  |
| *Aa* | Sensitivity | 0.15 | 0.05 − 0.36 |  | 0.75 | 0.53 − 0.89 |
|  | Specificity | 0.70 | 0.48 − 0.85 |  | 0.50 | 0.30 − 0.70 |
|  | Positive Predictive Value | 0.33 | 0.12 − 0.65 |  | 0.60 | 0.41 − 0.77 |
|  | Negative Predictive Value | 0.45 | 0.29− 0.62 |  | 0.67 | 0.42 − 0.85 |
|  |  |  |  |  |  |  |
|  |  |  |  |  |  |  |
| *Fn* | Sensitivity | 1.00 | 0.84 − 1.00 |  | 1.00 | 0.84 − 1.00 |
|  | Specificity | 0.00 | 0.00 − 0.15 |  | 0.00 | 0.00 − 0.15 |
|  | Positive Predictive Value | 0.50 | 0.35 − 0.65 |  | 0.50 | 0.35 − 0.65 |
|  | Negative Predictive Value |  |  |  |  |  |
|  |  |  |  |  |  |  |

*Pg*—*Porphyromonas gingivalis*; *Aa*—*Aggregatibacter actinomycetemcomitans*; *Fn*—*Fusobacterium nucleatum;* 95% CI—confidence interval of 95%.

**Supplementary Table 10.** Bland-Altman analysis of *Porphyromonas gingivalis*, *Aggregatibacter actinomycetemcomitans*, and *Fusobacterium nucleatum* quantification agreement from subgingival plaque samples.

|  |  |  |  |
| --- | --- | --- | --- |
| *Pg* | General | Bias, log_10_ Geq/mL (±SD) | -0.700 (1.650) |
|  |  | Limits of agreement, log_10_ Geq/mL | (-3.923−2.533) |
|  | Low bacterial load | Bias, log_10_ Geq/mL (±SD) | -1.850 (2.100) |
|  |  | Limits of agreement, log_10_ Geq/mL | (-5.966−2.266) |
|  | Medium bacterial load | Bias, log_10_ Geq/mL (±SD) | -0.001 (0.270) |
|  |  | Limits of agreement, log_10_ Geq/mL | (-0.533−0.522) |
|  | High bacterial load | Bias, log_10_ Geq/mL (±SD) | -0.068 (0.490) |
|  |  | Limits of agreement, log_10_ Geq/mL | (-0.889−1.026) |
|  |  |  |  |
|  |  |  |  |
| *Aa* | General | Bias, log_10_ Geq/mL (±SD) | 1.810 (1.930) |
|  |  | Limits of agreement, log_10_ Geq/mL | (-5.601−1.972) |
|  | Low bacterial load | Bias, log_10_ Geq/mL (±SD) | -2.103 (2.080) |
|  |  | Limits of agreement, log_10_ Geq/mL | (-6.171−1.964) |
|  | Medium bacterial load | Bias, log_10_ Geq/mL (±SD) | -1.000 (0.790) |
|  |  | Limits of agreement, log_10_ Geq/mL | (-2.541−0.542) |
|  | High bacterial load | Bias, log_10_ Geq/mL (±SD) | -0.461 (0.780) |
|  |  | Limits of agreement, log_10_ Geq/mL | (-1.991−1.070) |
|  |  |  |  |
|  |  |  |  |
| *Fn* | General | Bias, log_10_ Geq/mL (±SD) | 0.050 (0.360) |
|  |  | Limits of agreement, log_10_ Geq/mL | (-0.764−0.661) |
|  | Low bacterial load | Bias, log_10_ Geq/mL (±SD) | ND |
|  |  | Limits of agreement, log_10_ Geq/mL | ND |
|  | Medium bacterial load | Bias, log_10_ Geq/mL (±SD) | -0.652 (1.070) |
|  |  | Limits of agreement, log_10_ Geq/mL | (-2.753−1.449) |
|  | High bacterial load | Bias, log_10_ Geq/mL (±SD) | 0.015 (0.050) |
|  |  | Limits of agreement, log_10_ Geq/mL | (-0.092−0.122) |
|  |  |  |  |

Bland-Altman analysis results are subgrouped into arbitrary categories of low (< 3 log_10_), medium (3−6 log_10_), and high (> 6 log_10_ Geq/mL) bacterial load. *Pg*—*Porphyromonas gingivalis*; *Aa*—*Aggregatibacter actinomycetemcomitans*; Fn—*Fusobacterium nucleatum;* Geq—genome equivalents; SD—standard deviation; ND—not detected

**Supplementary Figure 1.** One-dimensional scatter plots from the optimization of the multiplex digital PCR assay showing the intensity of partition fluorescence at the acquisition channels when testing 10-fold serial dilutions of total DNA isolates of the bacterial reference strains *Aggregatibacter actinomycetemcomitans* (Aa DNA), *Porphyromonas gingivalis* (Pg DNA), and *Fusobacterium nucleatum* (Fn DNA) with an estimated DNA concentration of 10^4^, 10^3^, and 10^2^ copies/µL at an annealing temperature of 56°C, respectively. The primer-probe concentrations in the reaction mixtures were 0.4 µM of each of the primers and 0.2 µM of each of the probes **(A)**, and 0.9 µM of each of the primers and 0.25 µM of each of the probes **(B)**, respectively.


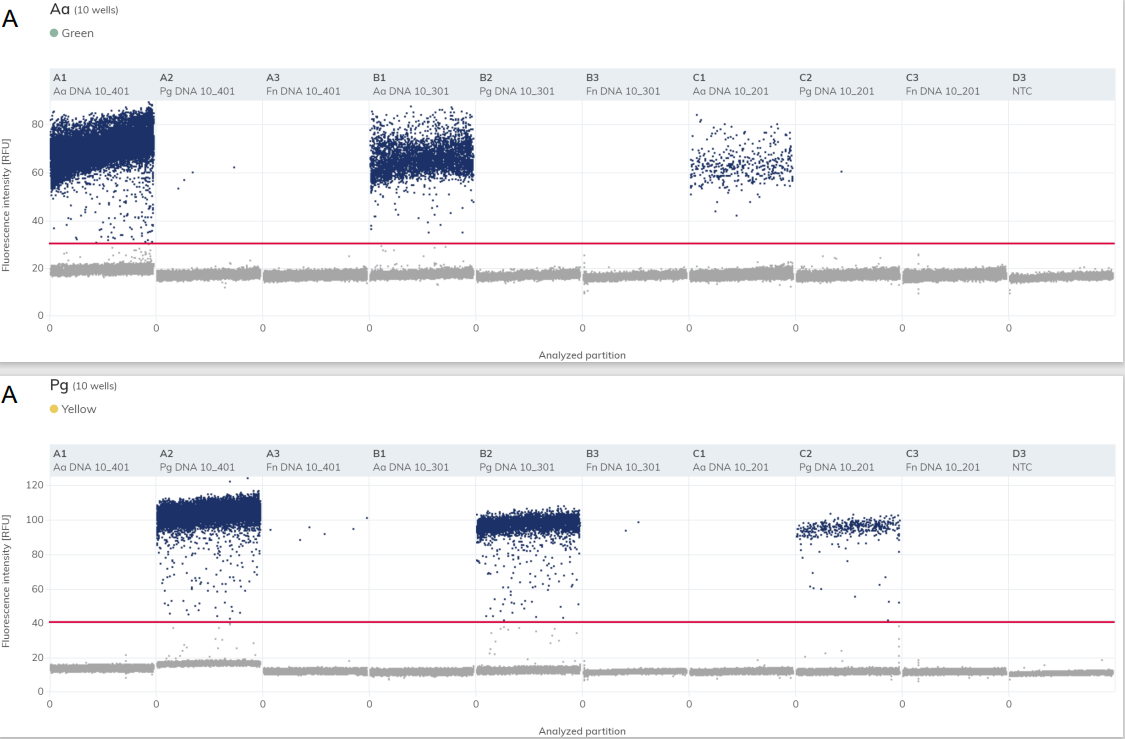

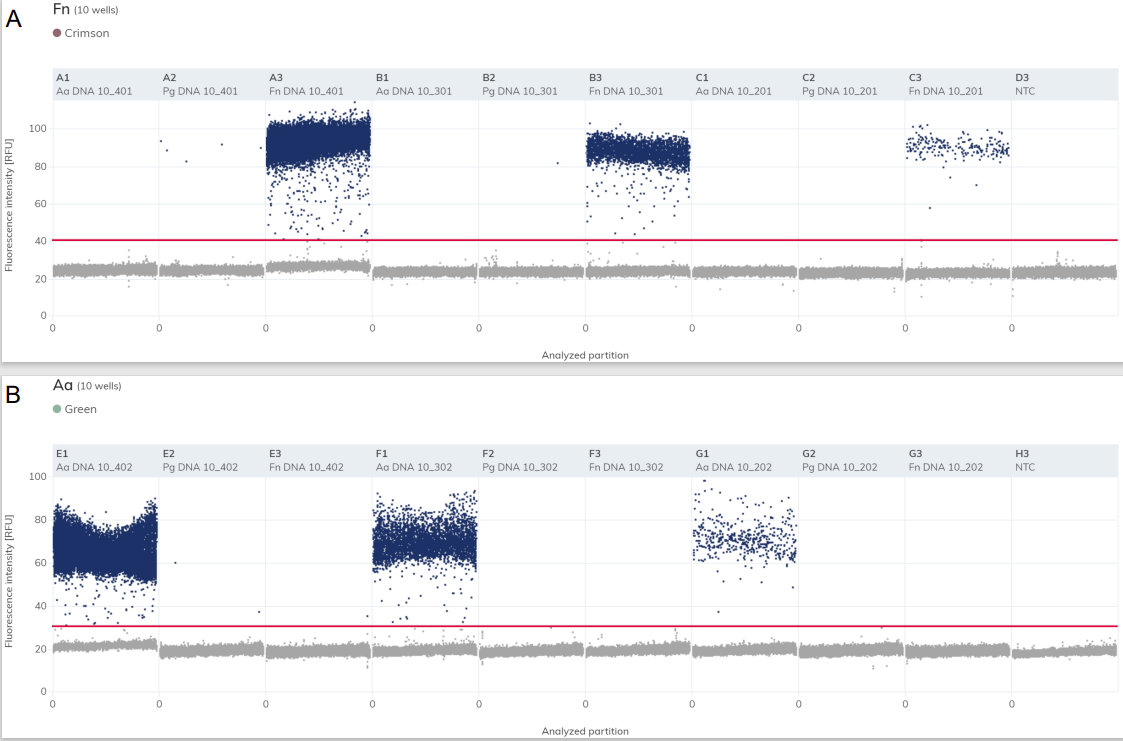


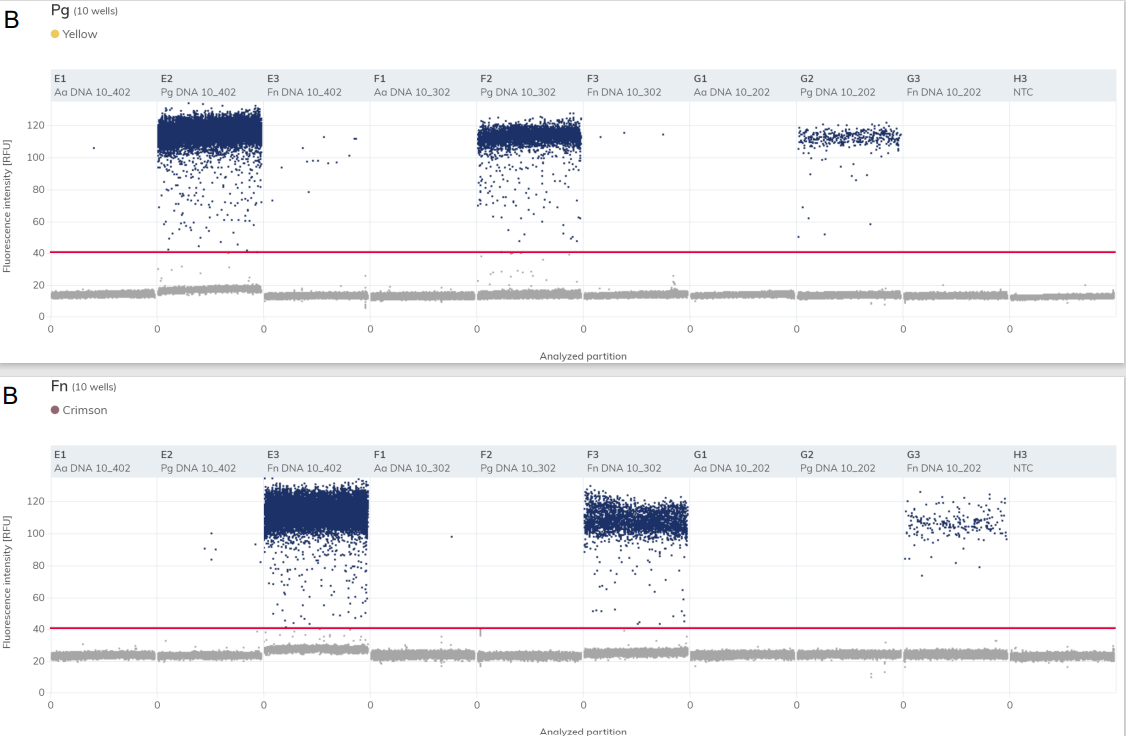


**Supplementary Figure 2.** One-dimensional scatter plots from the optimization of the multiplex digital PCR assay showing the intensity of partition fluorescence at the acquisition channels when testing 10-fold serial dilutions of total DNA isolates of the bacterial reference strains *Aggregatibacter actinomycetemcomitans* (Aa DNA), *Porphyromonas gingivalis* (Pg DNA), and *Fusobacterium nucleatum* (Fn DNA) with an estimated DNA concentration of 10^4^, 10^3^, and 10^2^ copies/µL at an annealing temperature of 58°C, respectively. The primer-probe concentrations in the reaction mixtures were 0.4 µM of each of the primers and 0.2 µM of each of the probes **(A)**, and 0.9 µM of each of the primers and 0.25 µM of each of the probes **(B)**, respectively.


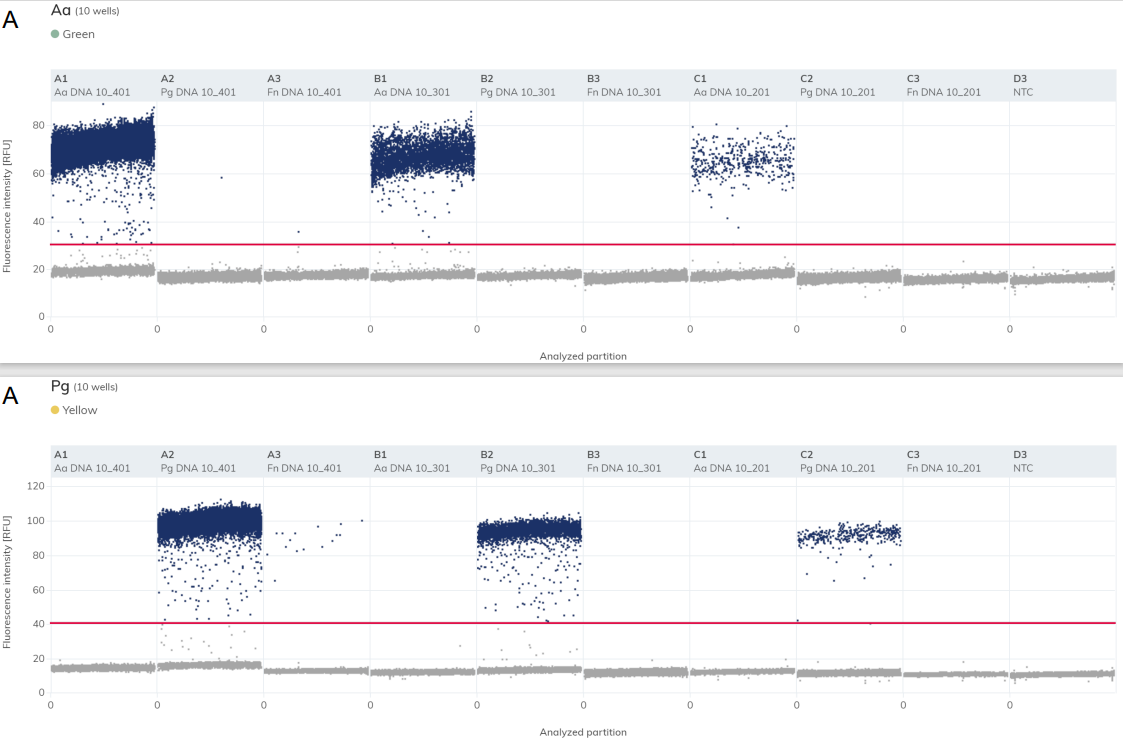

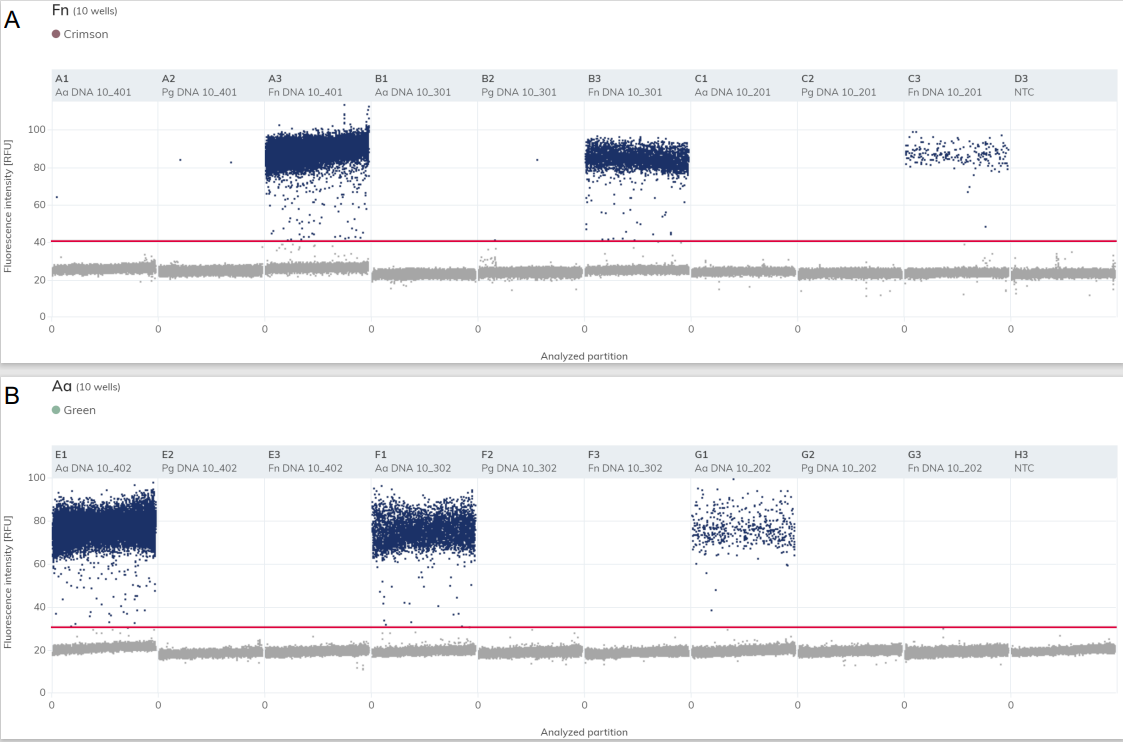

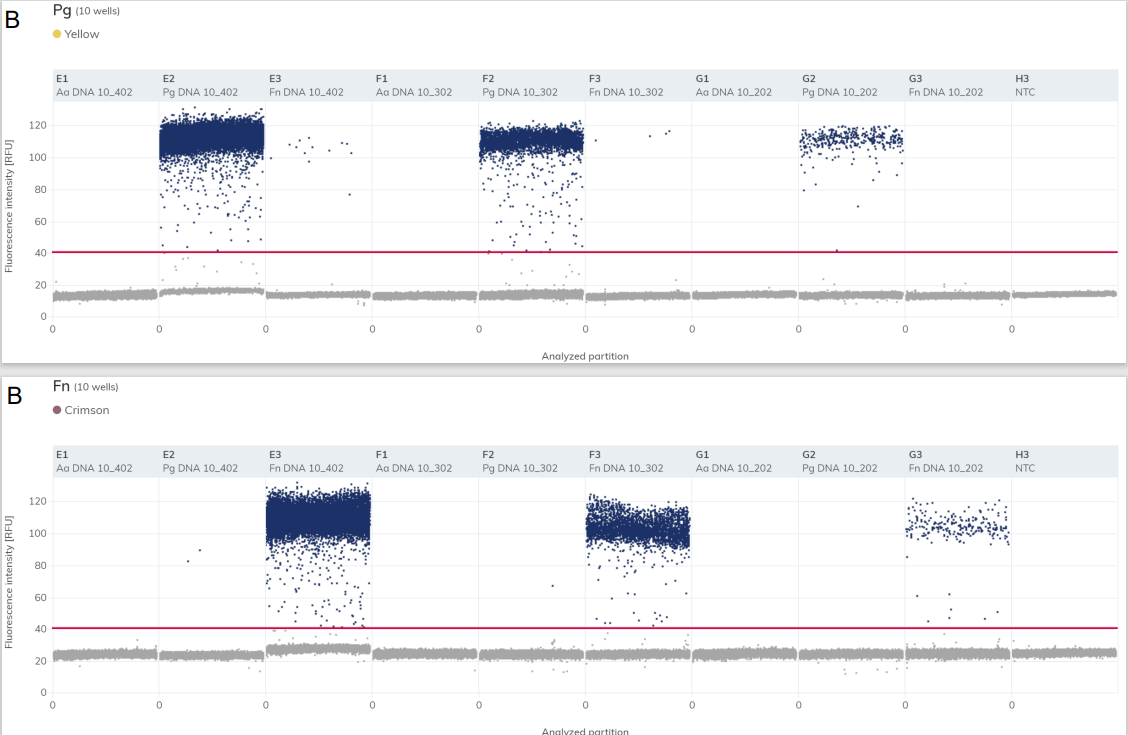


**Supplementary Figure 3.** One-dimensional scatter plots from the optimization of the multiplex digital PCR assay showing the intensity of partition fluorescence at the acquisition channels when testing 10-fold serial dilutions of total DNA isolates of the bacterial reference strains *Aggregatibacter actinomycetemcomitans* (Aa DNA), *Porphyromonas gingivalis* (Pg DNA), and *Fusobacterium nucleatum* (Fn DNA) with an estimated DNA concentration of 10^4^. 10^3^. and 10^2^ copies/µL at an annealing temperature of 60°C. The primer-probe concentrations in the reaction mixtures were 0.4 µM of each of the primers and 0.2 µM of each of the probes **(A)**, and 0.9 µM of each of the primers and 0.25 µM of each of the probes **(B)**, respectively. In the test with an annealing temperature of 60°C, only the first combination of primer-probe concentration was used.


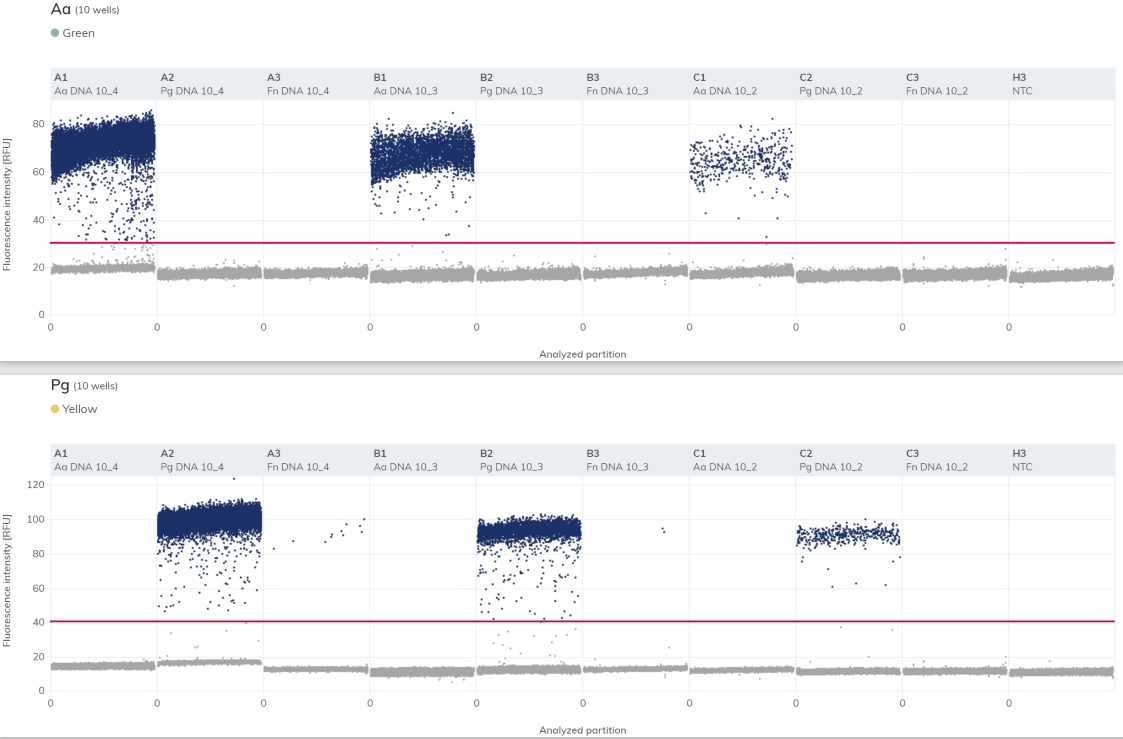

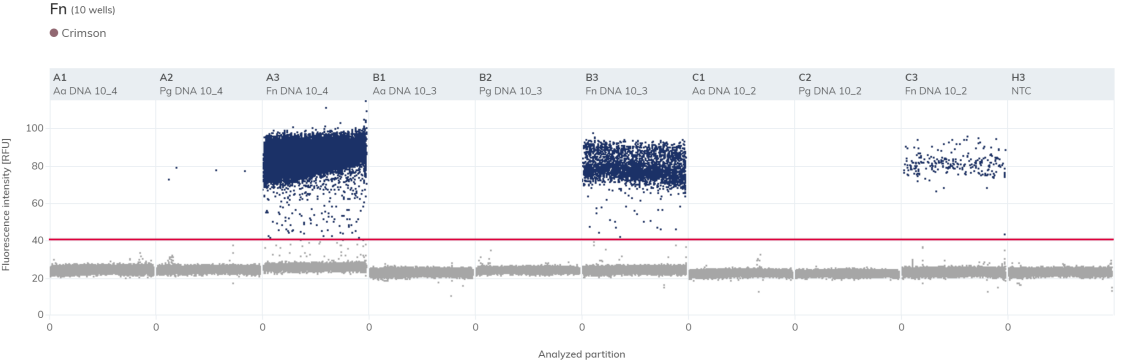


**Supplementary Figure 4.** One-dimensional scatter plots from the optimization of the multiplex digital PCR assay showing the intensity of partition fluorescence at the acquisition channels when testing 10-fold serial dilutions of total DNA isolates of the bacterial reference strains *Aggregatibacter actinomycetemcomitans* (Aa DNA), *Porphyromonas gingivalis* (Pg DNA), and *Fusobacterium nucleatum* (Fn DNA) with an estimated DNA concentration of 10^4^, 10^3^, and 10^2^ copies/µL at an annealing temperature of 62°C. The primer-probe concentrations in the reaction mixtures were 0.4 µM of each of the primers and 0.2 µM of each of the probes **(A)**, and 0.9 µM of each of the primers and 0.25 µM of each of the probes **(B)**, respectively.


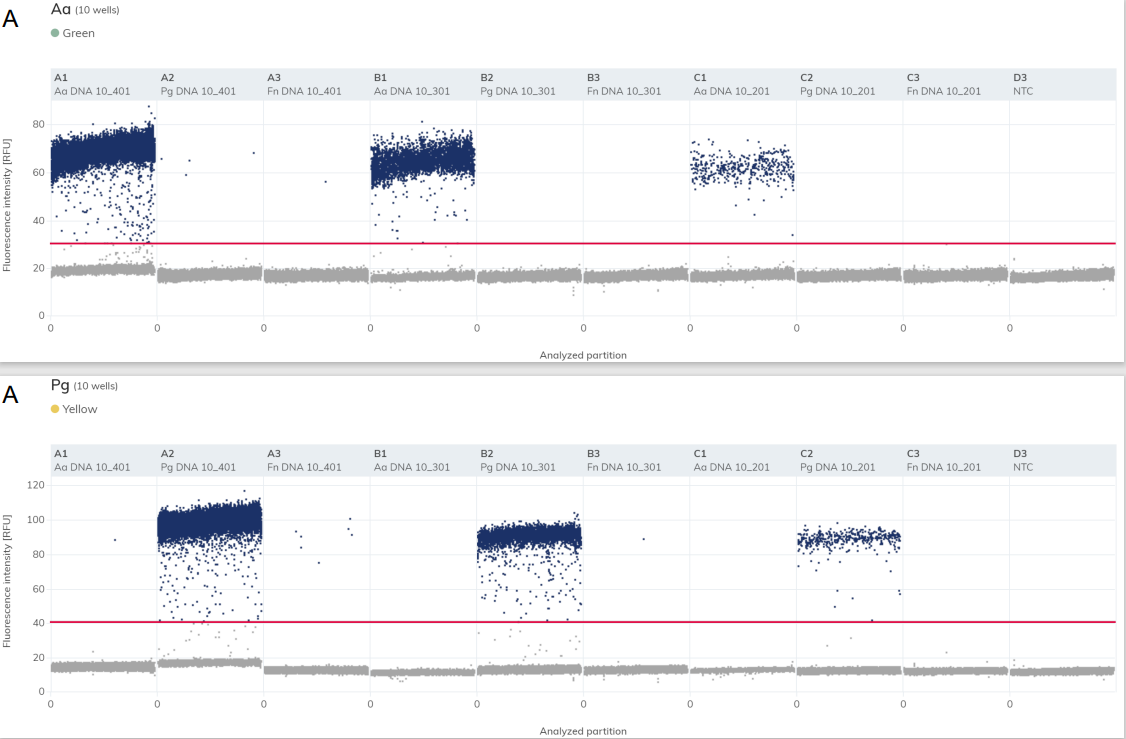

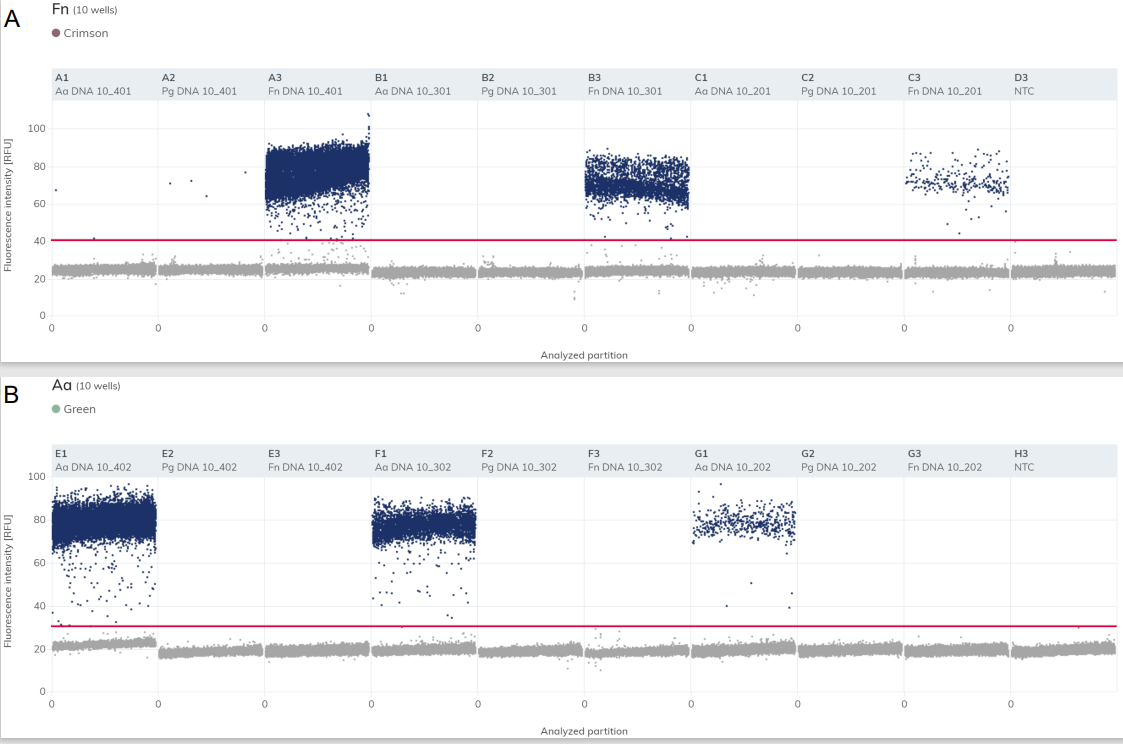

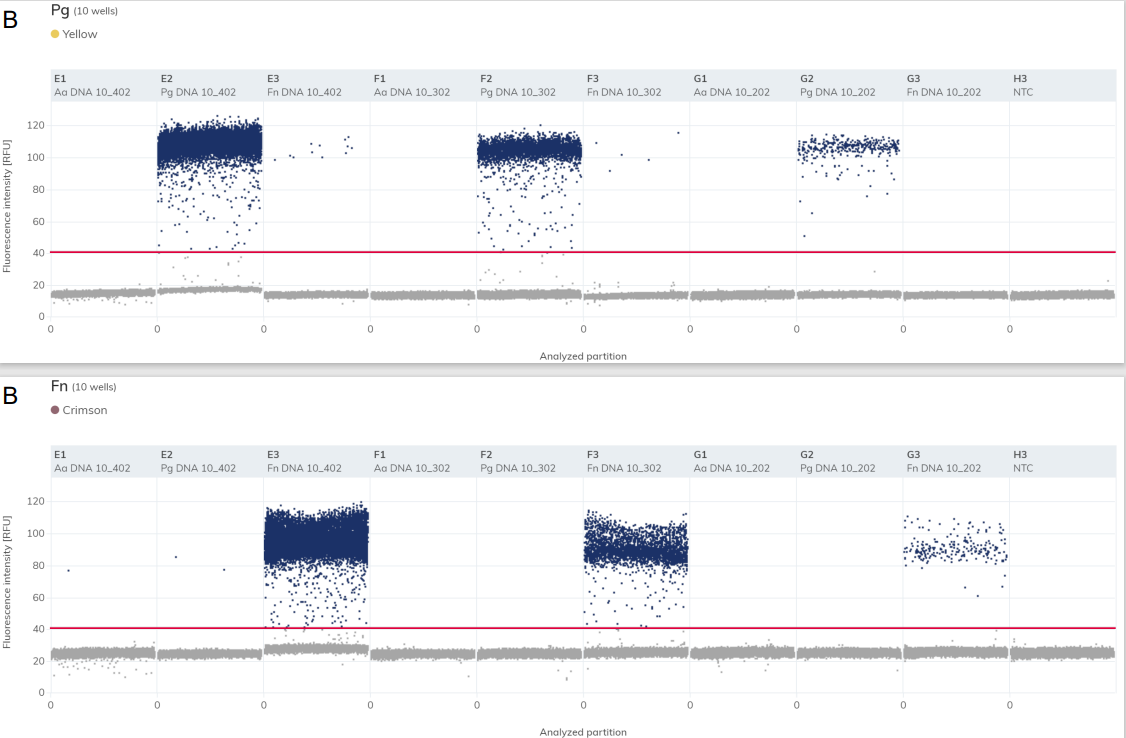


**Supplementary Figure 5.** Exemplary one-dimensional scatter plots showing the intensity of partition fluorescence at the acquisition channels when testing total DNA isolates of the clinical samples and the no-template control (NTC) with the multiplex digital PCR assay.


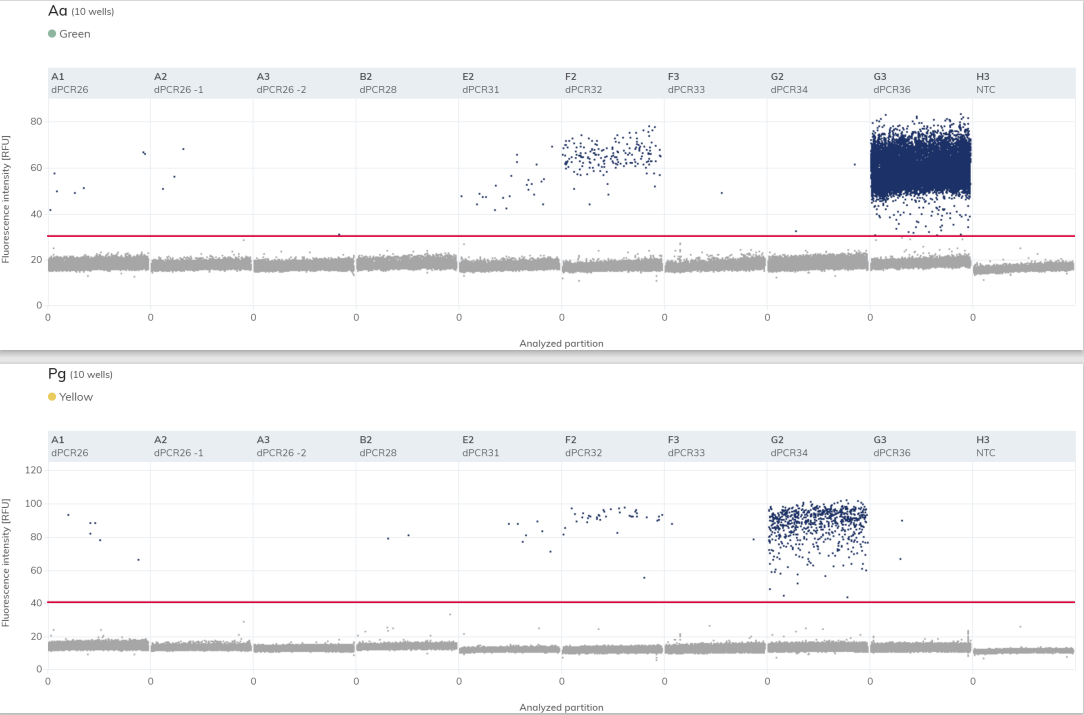


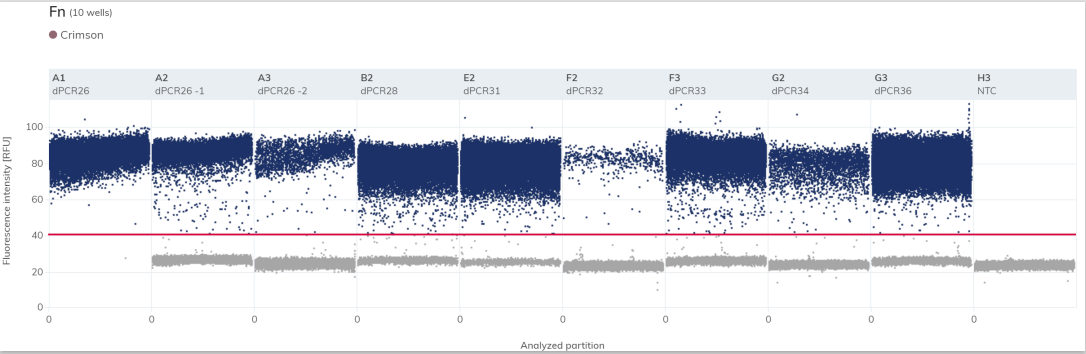


**Supplementary Figure 6.** Linear regressions between the measured and estimated concentration of bacterial target DNA of *Porphyromonas gingivalis*, *Aggregatibacter actinomycetemcomitans*, and *Fusobacterium nucleatum* over the dynamic range of the multiplex quantitative real-time PCR and digital PCR assays.


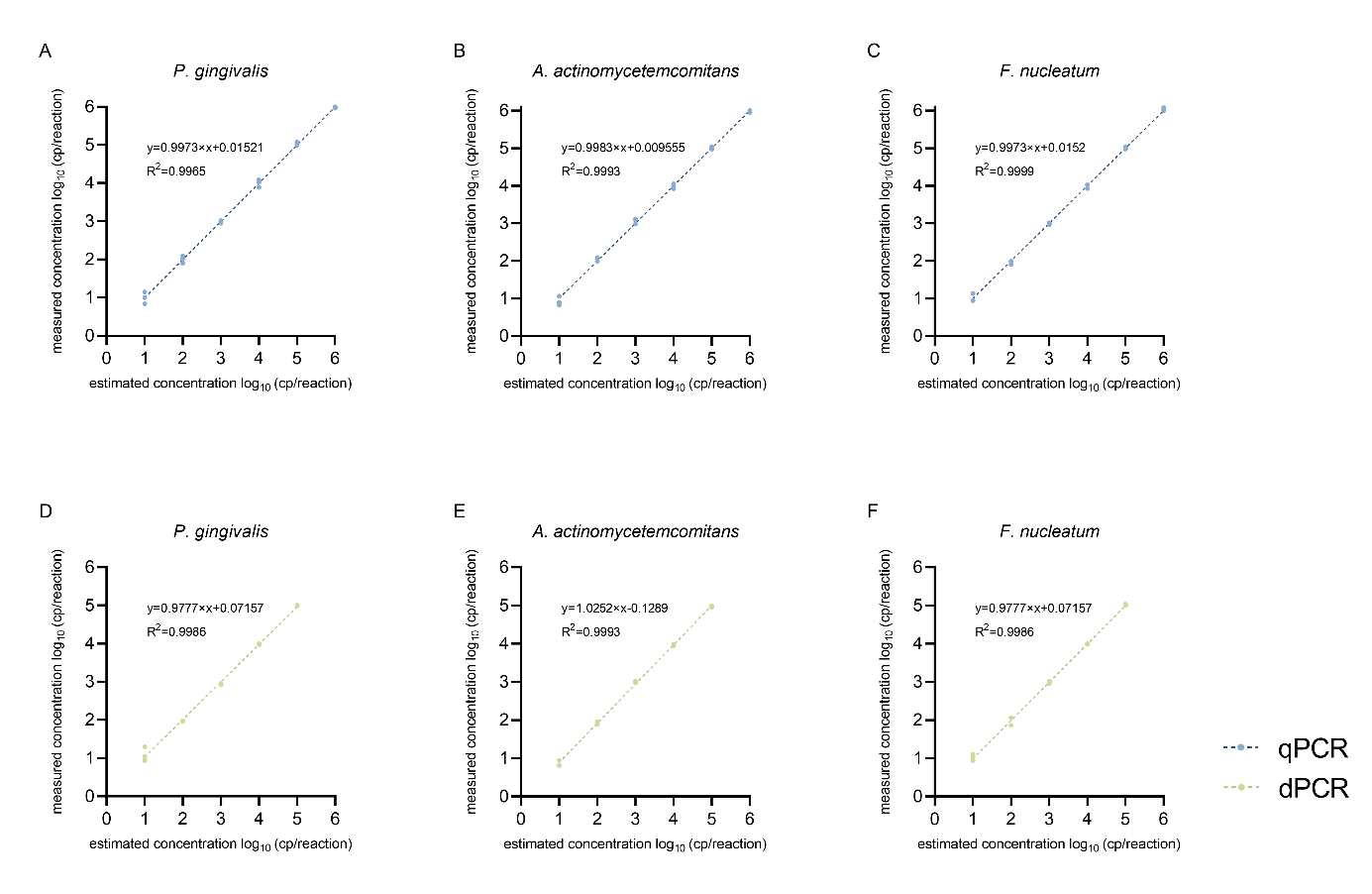


Linear regression of the multiplex quantitative real-time PCR (qPCR) and digital PCR (dPCR) assays between measured and estimated concentration of bacterial target DNA (in log_10_ copies per reaction). Multiplex qPCR assay — (**A)** *Porphyromonas gingivalis,* **(B)** *Aggregatibacter actinomycetemcomitans,* and **(C)** *Fusobacterium nucleatum*. Multiplex dPCR assay — (**D)** *Porphyromonas gingivalis,* **(E)** *Aggregatibacter actinomycetemcomitans,* and **(F)** *Fusobacterium nucleatum*. cp—copie

**Supplementary Figure 7.** One-dimensional scatter plots showing the intensity of partition fluorescence at the acquisition channels when testing triplicates of 10-fold serially diluted standards of bacterial target DNA, corresponding to the estimated input concentration range of 1 × 10^6^ to 1 × 10^−1^ copies/reaction with the multiplex digital PCR assay **(A, C, and E)** and the singleplex digital PCR assays targeting *Aggregatibacter actinomycetemcomitans* **(B)**, *Porphyromonas gingivalis* **(D)**, and *Fusobacterium nucleatum* **(F)**, respectively.


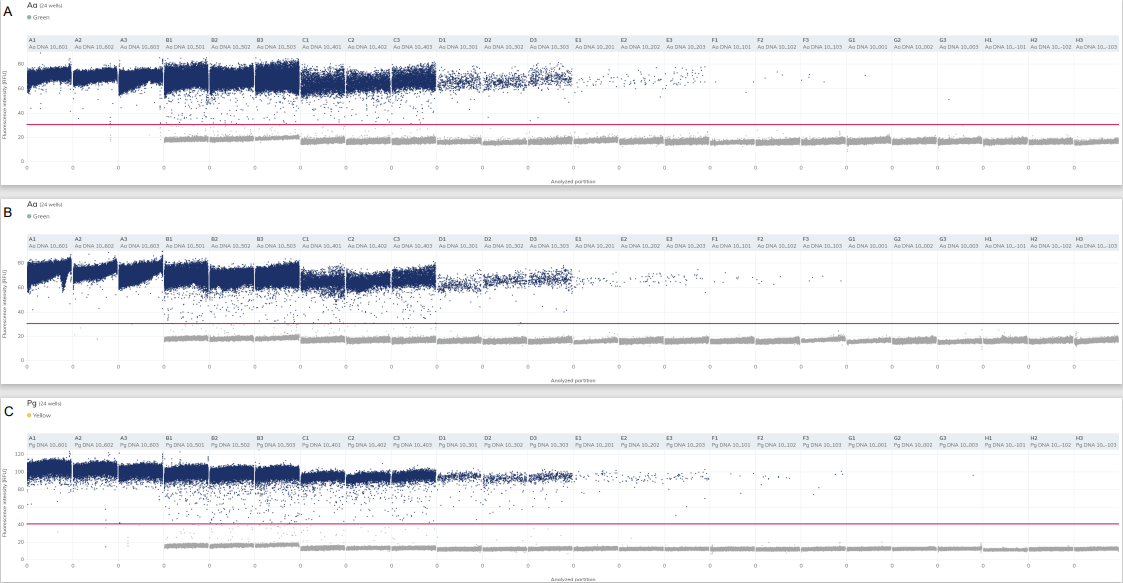

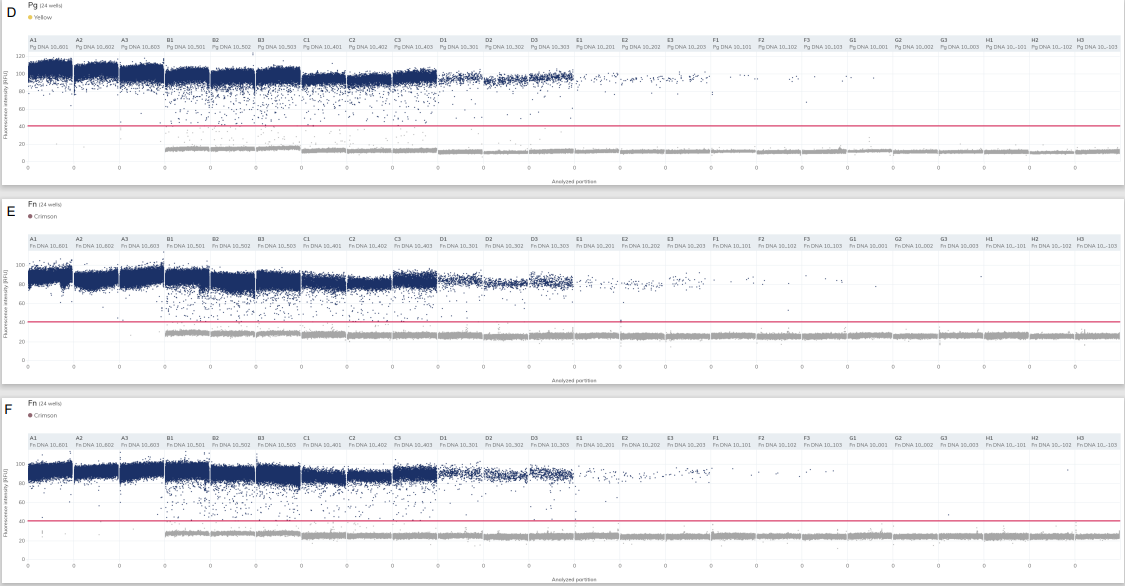


**Supplementary Figure 8.** Precision of quantification of bacterial target DNA in the serially diluted standards of *Porphyromonas gingivalis*, *Aggregatibacter actinomycetemcomitans*, and *Fusobacterium nucleatum* by the singleplex quantitative real-time PCR and digital PCR assays.


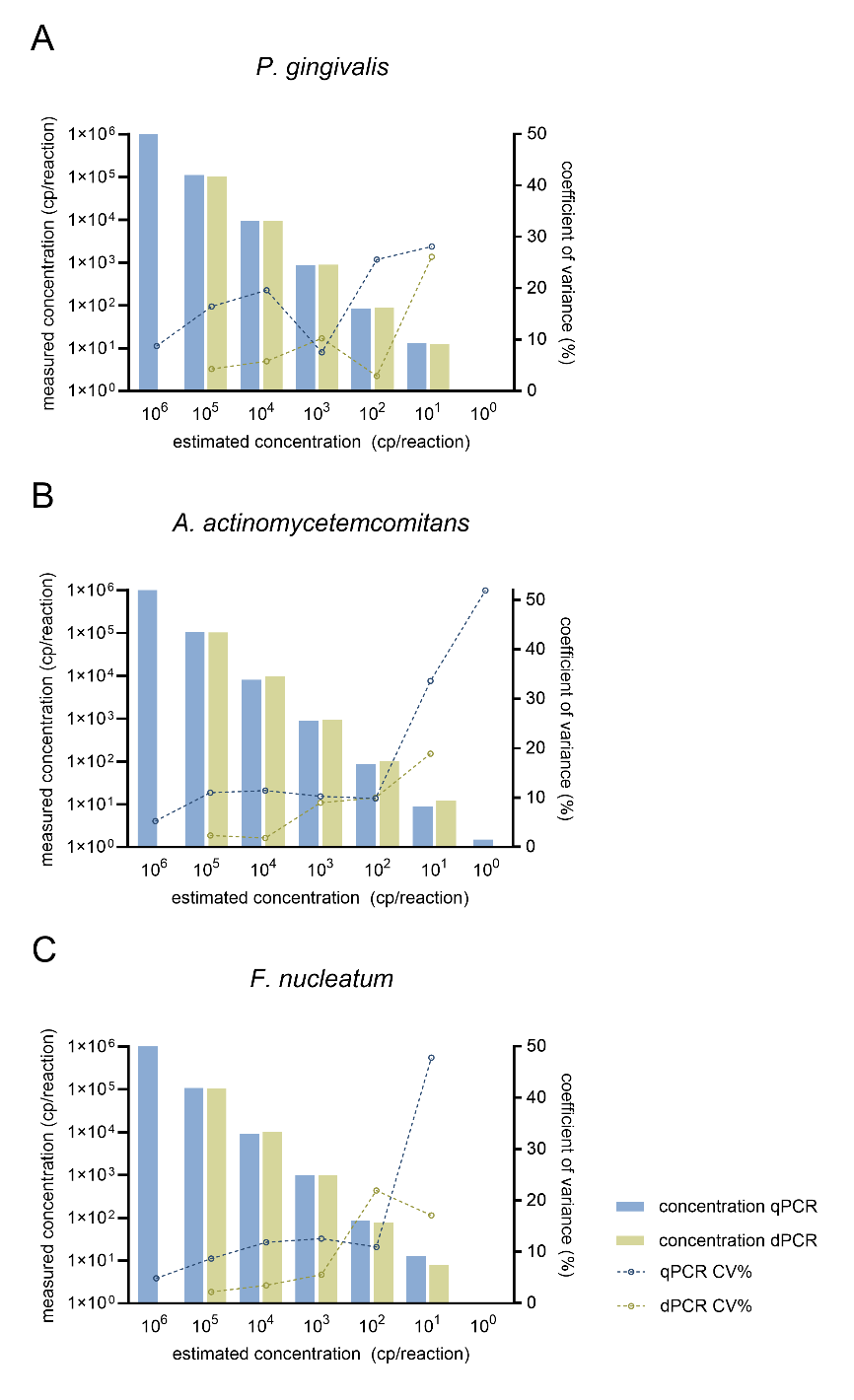


The precision (intra-assay variability) of the singlplex quantitative real-time PCR (qPCR) and digital PCR (dPCR) assays was determined by assessing the coefficient of variation percentage (CV%) in the quantification of bacterial target DNA (in copies per reaction) in serial dilutions of the standards of **(A)** *Porphyromonas gingivalis,* **(B)** *Aggregatibacter actinomycetemcomitans,* and **(C)** *Fusobacterium nucleatum*.

**Supplementary Figure 9.** One-dimensional scatter plots showing the intensity of partition fluorescence at the acquisition channels when testing human genomic DNA and no-template control (NTC) with the multiplex digital PCR assay (wells A1 and B1) and the singleplex digital PCR assays targeting *Aggregatibacter actinomycetemcomitans* (wells C1 and D1), *Porphyromonas gingivalis* (wells E1 and F1), and *Fusobacterium nucleatum* (wells G1 and H1), respectively.


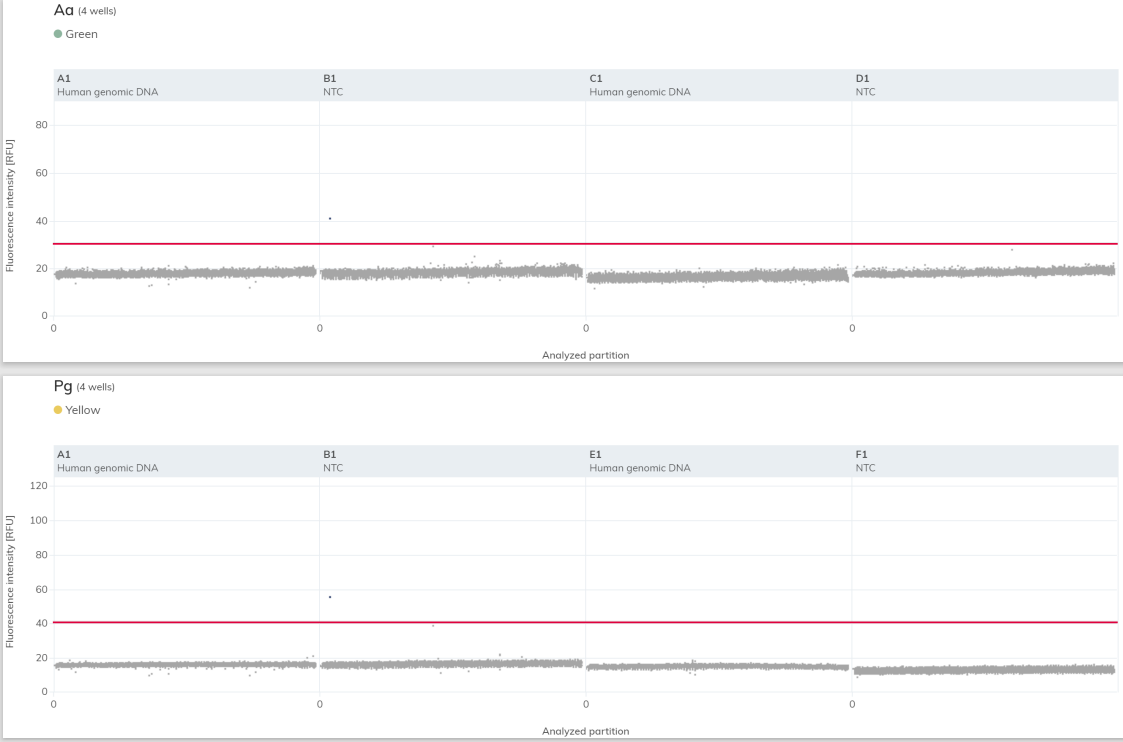

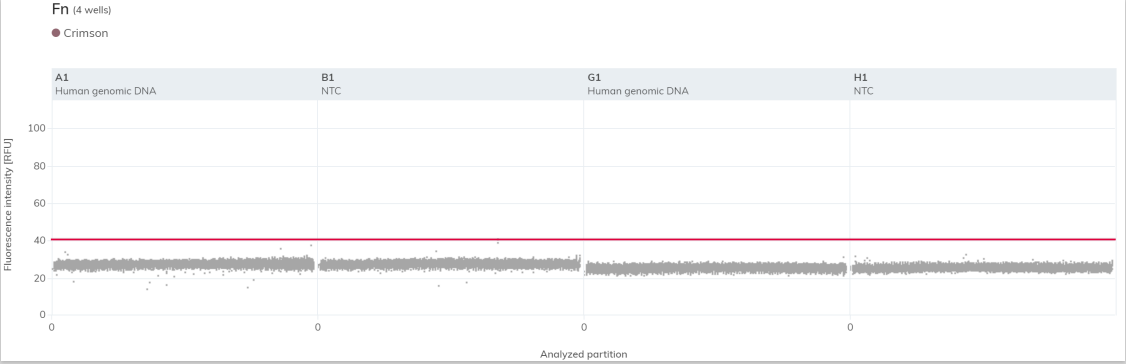


**References**

1. Katoh K. Standley DM. MAFFT Multiple Sequence Alignment Software Version 7: Improvements in Performance and Usability. *Mol Biol Evol*. 2013;30(4):772-780. doi:10.1093/molbev/mst010

2. Koressaar T. Remm M. Enhancements and modifications of primer design program Primer3. *Bioinformatics*. 2007;23(10):1289-1291. doi:10.1093/bioinformatics/btm091

3. Untergasser A. Cutcutache I. Koressaar T. et al. Primer3—new capabilities and interfaces. *Nucleic Acids Res*. 2012;40(15):e115-e115. doi:10.1093/nar/gks596

4. Zayed N. Munjaković H. Aktan MK. et al. Electrolyzed Saline Targets Biofilm Periodontal Pathogens In Vitro. *J Dent Res*. 2024;103(3):243-252. doi:10.1177/00220345231216660
